# Global identification and analysis revealed differentially expressed lncRNAs associated with meiosis and low fertility in autotetraploid rice

**DOI:** 10.1186/s12870-020-2290-0

**Published:** 2020-02-19

**Authors:** Xiang Li, Muhammad Qasim Shahid, Minsi Wen, Shuling Chen, Hang Yu, Yamin Jiao, Zijun Lu, Yajing Li, Xiangdong Liu

**Affiliations:** 1 State Key Laboratory for Conservation and Utilization of Subtropical Agro-Bioresources, South China Agricultural University, Guangzhou, 510642 China; 2 Guangdong Provincial Key Laboratory of Plant Molecular Breeding, South China Agricultural University, Guangzhou, 510642 China; 3 College of Agriculture, South China Agricultural University, Guangzhou, 510642 China; 4 Guangdong Laboratory for Lingnan Modern Agriculture, South China Agricultural University, Guangzhou, 510642 China

**Keywords:** Autotetraploid rice, Embryo sac mother cell, lncRNAs (long non-coding RNAs), Male sterility, Meiosis, Pollen mother cell

## Abstract

**Background:**

Autotetraploid rice is a useful germplasm for polyploid rice breeding. Our previous research showed that non-coding RNAs might be associated with low fertility in autotetraploid rice. However, little information is available on long non-coding RNAs (lncRNAs) involved in the low fertility of autotetraploid rice. In the present study, RNA-seq was employed to detect the differentially expressed meiosis-related lncRNAs in autotetraploid rice, and gene overexpression and knock out experiments were used to validate the potential function of candidate lncRNA.

**Results:**

A total of 444 differentially expressed lncRNAs (DEL) were detected during anther and ovary meiosis in autotetraploid rice. Of these, 328 DEL were associated with the transposable elements, which displayed low expression levels during meiosis in autotetraploid rice. We used rapid amplification of cDNA ends (RACE) assay to validate 10 DEL and found that the lncRNAs were not assembly artifacts, and six of them were conserved in tetraploid rice. Moreover, 237 and 20 lncRNAs were associated with pollen mother cell (PMC) and embryo sac mother cell (EMC) meiosis in autotetraploid rice, respectively. The differential expressions of some meiosis-related targets and its DEL regulator, including *MEL1* regulated by TCONS_00068868, *LOC_Os12g41350* (meiotic asynaptic mutant 1) by TCONS_00057811 in PMC, and *LOC_Os12g39420* by TCONS_00144592 in EMC, were confirmed by qRT-PCR. TCONS_00057811, TCONS_00055980 and TCONS_00130461 showed anther specific expression patterns and were found to be highly expressed during meiosis. CRISPR/Cas9 editing of lncRNA57811 displayed similar morphology compared to wild type. The overexpression of lncRNA57811 resulted in low pollen fertility (29.70%) and seed setting (33%) in rice.

**Conclusion:**

The differential expression levels of lncRNAs, associated with transposable elements and meiosis-regulated targets, might be endogenous noncoding regulators of pollen/embryo sac development that cause low fertility in autotetraploid rice. The results enhance our understanding about rice lncRNAs, and facilitate functional research in autotetraploid rice.

## Background

Whole genome doubling (WGD), or polyploidy, has been known as a vital evolutionary force for genetic diversity in plants. About 35%~ 70% angiosperms have undergone at least one round of genome duplication, such as wheat, cotton and *brassica* [1]. A variety of genome alterations caused by polyploidy, including creating new source of redundant genes, genome rearrangements and genome-wide subfunctionalization and neofunctionalization [2, 3]. The potential advantages of plant genome reconstruction can be utilized with the newly developed resources of genomics for crop improvement [4].

Autotetraploid rice, as a useful germplasm, has more biological advantages than the diploid rice that may become a new way for rice breeding [5–7]. However, low fertility, an unfavorable trait, is one of the major barriers in commercial production of polyploid rice [8–11]. Meiosis is an essential biological process in the life cycle of sexual reproduction in plants [12]. Normal chromosomes segregation in meiosis is one of the most important challenges to polyploids [13, 14]. Multivalents during meiosis often generated massively in the newly formed polyploids [15–18]. Cytogenetic analysis revealed that chromosomal abnormalities, abnormal development processes of pollens and embryo sacs were the main reasons for low fertility in autotetraploid rice [16, 19, 20]. Transcriptome analysis revealed that polyploidy enhances the F_1_ pollen sterility multi-loci interactions which lead to meiosis abnormalities and pollen sterility in autotetraploid rice hybrids [19]. In addition, some meiosis-related microRNAs were identified that may interrupt the chromosome behavior in pollen mother cells (PMC) of autotetraploid rice [21]. Recently, differential expression patterns of small RNAs during embryo sac development in autotetraploid rice have been reported to associate with sterility, especially during megasporocyte [20].

Non-coding RNAs (ncRNAs) cannot encode proteins; however, they play a key role in regulation of transcriptional, posttranscriptional and epigenetic mechanisms. Long non-coding RNAs (lncRNAs) are one of the important types of ncRNAs, with a length of more than 200 nt, and usually exhibited tissue/developmental-stage-specific expression patterns [22]. LncRNAs have been identified in many plants, including rice, maize, sunflower and strawberry [23–25], and some of them were reported to associate with reproductive processes in rice [26]. Additionally, the lncRNAs of rice anthers before flowering, pistils before flowering, spikelets 5 days after pollination and shoots 14 days after germination have been characterized, and several important lncRNAs were exclusively identified in young panicles, which showed a profound impact on rice fertility [27]. Recently, overexpression of *LAIR* increased grain yield and up-regulated the expression of several leucine-rich repeat receptor kinase genes in rice [28]. Meanwhile, lncRNAs played a pivotal role to small RNAs population, which could act as small RNAs precursors, miRNAs targets and decoys [29].

Although information on small RNAs and transcriptome sequencing in autotetraploid rice are available [11, 20], the expression patterns of lncRNAs associated with meiosis in autotetraploid rice are still poorly understood. The present study was planned to investigate the expression levels of lncRNAs in anther and ovary during meiosis in an autotetraploid rice, Taichung 65-4x, and its diploid counterpart (Taichung 65) by high-throughput sequencing. We sought to distinguish the lncRNAs changes between autotetraploid and diploid rice, and to determine the relationships between gene expression profiles and spikelet sterility.

## Results

### Identification and characterization of lncRNAs in rice anther and ovary

**Fig. 1:**
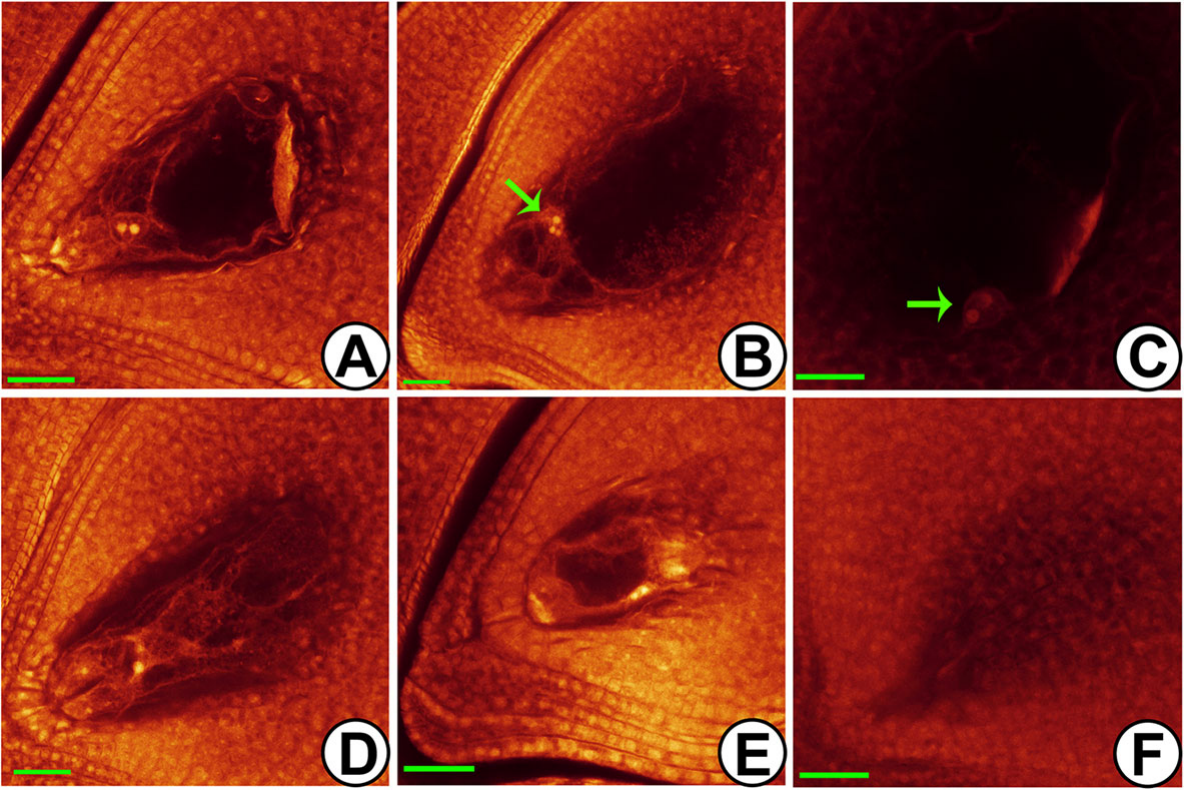
Cytological observation of embryo sac in Taichung 65-4x by WE-CLSM. **a** normal embryo sac. **b**, **c** abnormal position and number of polar nuclei (green arrow). **d** abnormal antipodal cells. **e** small embryo sac. **f** embryo sac degeneration. Bars = 40 μm

Previous study showed that the pollen fertility of Taichung 65-4x was lower than the diploid counterpart [11]. Here, embryo sac fertility of Taichung 65-4x was observed by Whole-mount eosin B-staining confocal laser scanning microscopy (Fig. 1), which was 62.15%. Therefore, anthers and corresponding ovaries of Taichung 65 and Taichung 65-4x from pre-meiotic interphase to meiosis prophase I (early meiosis stage) were collected for transcriptome sequencing. After a genome-wide systematic filtration, a total of 4859 confident lncRNAs (FPKM > 0.5 in at least one library) were obtained, including 4018 intergenic lncRNAs, 790 antisense lncRNAs, and 51 sense lncRNAs, and used for further analysis (Additional file 1: Fig. S1A; Additional file 2: Table S1). Venn analysis revealed that most of the lncRNAs were specifically expressed in anthers/ovaries, and a total of 1282 and 2025 lncRNAs were specifically detected in the rice reproductive tissues of Taichung 65 and Taichung 65-4x, respectively (Additional file 1: Fig. S1B). Moreover, 28% lncRNAs were exclusively expressed in the anthers of Taichung 65-4x.

We further characterized the features of lncRNAs, and found that the expression levels of lncRNAs were lower than the protein-coding genes (Additional file 1: Fig. S2A). High proportions of lncRNAs were found with one exon, which differed from the protein-coding genes (Additional file 1: Fig. S2B). The transcript length distribution was not similar between lncRNAs and protein-coding genes. Most of protein-coding genes were longer than 1 kb, whereas majority of lncRNAs were less than 1 kb. The lengths of the lncRNAs, with a mean value of 938 nucleotides, was shorter than the protein-coding genes (mean = 1862 bp) (Additional file 1: Fig. S2C). In addition, shorter ORFs (open reading frames) were observed in lncRNAs (mean = 66.36 aa) than protein-coding genes (mean = 384.27 aa) in the rice reproductive tissues (Additional file 1: Fig. S2D). Moreover, only a small portion (4.4%) of rice reproductive lncRNAs was found to be conserved, including 12 in *Arabidopsis*, 80 in *Brachypodium*, 103 in *Zea mays*, and 161 in *Sorghum bicolor*. Intriguingly, 11 of 12 conserved lncRNAs in *Arabidopsis* were also found to be conserved in *Brachypodium*, *Zea mays* and *Sorghum bicolor* (Additional file 2: Table S2).

**Fig. 2:**
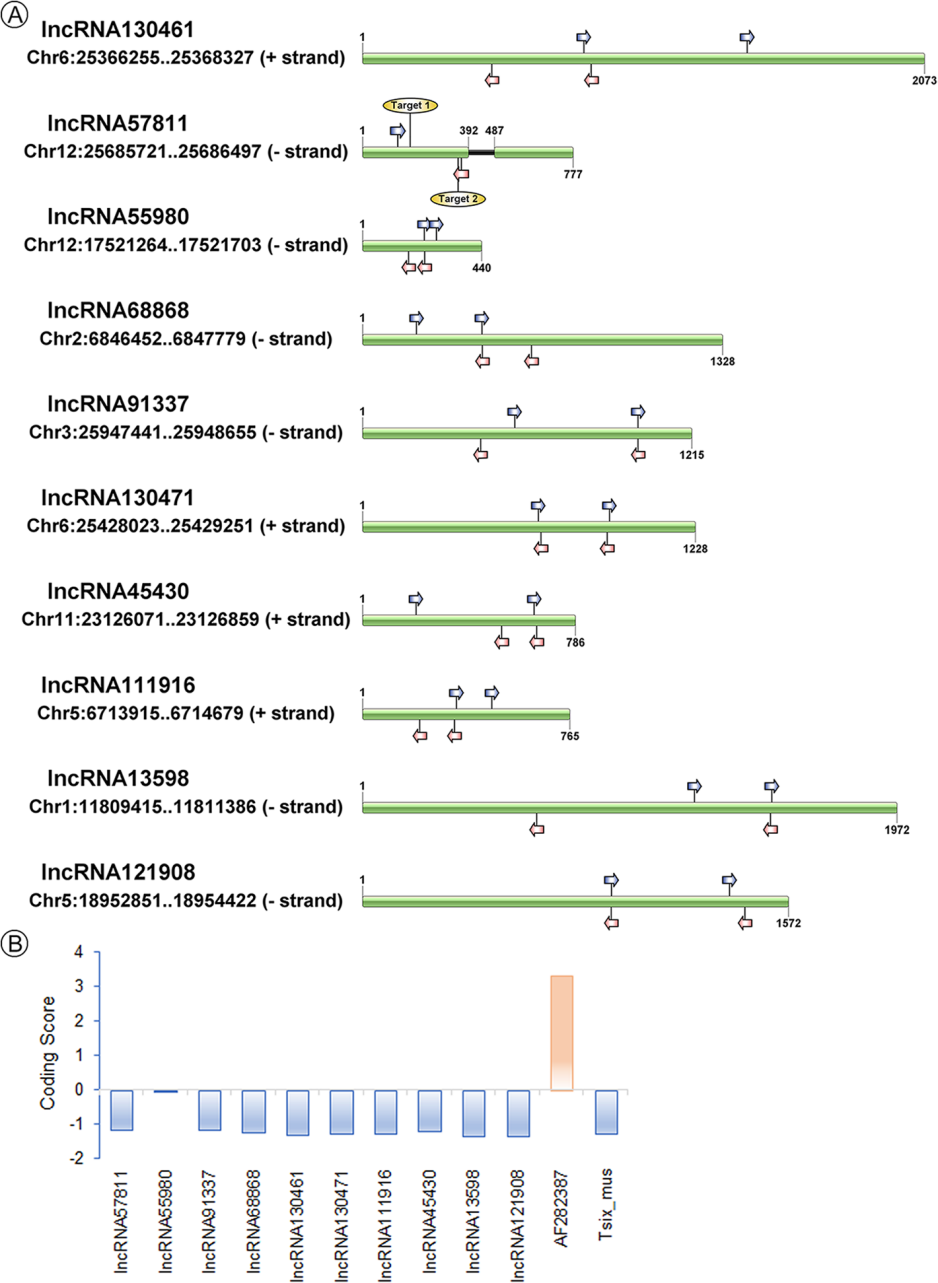
Features of the ten lncRNAs validation by RACE assay. **a** Structures of the lncRNAs. Blue and red arrows indicated the 3′ Race outer/inner primers and 5′ Race outer/inner primers, respectively. Target 1 and Target 2 represent the position of CRISPR/Cas 9 target sequence. The full-length of lncRNAs was drawn by IBS software (http://ibs.biocuckoo.org/). **b** Analysis of the coding potential of the ten lncRNAs. Transcripts with scores <−1 and > 1 are marked as non-coding or coding in CPC analysis [30]. AF282387 and Tsix_mus are provided as coding examples and non-coding examples by CPC analysis

The full-length of ten lncRNAs were obtained by rapid amplification of cDNA ends (RACE) assay (Fig. 2), including TCONS_00057811, TCONS_00130461, TCONS_00068868, TCONS_00091337, TCONS_00055980, TCONS_00130471, TCONS_00111916, TCONS_00045430, TCONS_00013598 and TCONS_00121908. These lncRNAs could be amplified and existed in the rice which indicated that lncRNAs were not assembly artifacts; however, no DNA variations were found between Taichung 65 and Taichung 65-4x. TCONS_00057811 was located in intergenic region (25685721–25,686,497, − strand) on chromosome 12. The real length of TCONS_00057811 was 777 bp, and it had two exons, which was different from the gene body structure predicted by transcriptome analysis. The length of TCONS_00130461 was 2073 bp, which was also different from the transcriptome result. Not only TCONS_00057811 and TCONS_00130461, the length of the other eight lncRNAs were different from the predicted fragments (Additional file 1: Fig. S3). Furthermore, phylogenetic tree (MEGA-X) results showed that six lncRNAs were conserved among our rice dataset (re-sequencing data of more than 100 rice lines, including neo-tetraploid, autotetraploid, typical *japonica*/*indica* and wild rice lines, and unpublished data), including TCONS_00057811, TCONS_00091337, TCONS_00130461, TCONS_00130471, TCONS_00045430 and TCONS_00055980 (Additional file 1: Fig. S4).

### Differentially expressed lncRNAs in anther and ovary during meiosis

To characterize the differential expression patterns of lncRNAs, we first figured out the lncRNAs associated with anther and ovary. By cluster analysis, 78 anther-preferred and 222 ovary-preferred lncRNAs, which highly/specifically expressed in anther/ovary, were identified in Taichung 65 (defined as anther- and ovary-preferred-2x), and 222 anther-preferred and 394 ovary-preferred lncRNAs were detected in Taichung 65-4x (defined as anther- and ovary-preferred-4x), respectively (Additional file 1: Fig. S5; Additional file 2: Table S1). Of the preferred lncRNAs in ovary, 133 lncRNAs were detected in both Taichung 65 and Taichung 65-4x during meiosis (co-ovary-preferred), and 34 lncRNAs were shared by the Taichung 65 and Taichung 65-4x in anther during meiosis (co-anther-preferred).

A total of 431 differentially expressed lncRNAs (DEL) were detected in anthers of Taichung 65-4x compared to the Taichung 65 (DEL-anther), of these 221 and 210 lncRNAs showed up- and down-regulation, respectively (Additional file 2: Table S3). Besides, a small portion of DEL (32 in total) was also found in ovaries of Taichung 65-4x compared to Taichung 65 (DEL-ovary), and 8 and 24 lncRNAs exhibited up- and down-regulation, respectively. 19 common DEL were detected between DEL-anther and DEL-ovary by venn analysis, and 17 out of these showed similar differential expression patterns with 3 co-up-regulated and 14 co-down-regulated DEL in autotetraploid rice (Additional file 1: Fig. S6). Furthermore, we compared the 431 DEL-anther and 32 DEL-ovary with the anther-preferred lncRNAs and ovary-preferred lncRNAs by venn analysis, respectively. 27 out of 34 co-anther-preferred lncRNAs showed differential expression patterns in Taichung 65-4x (DEL-anther: type 1). Surprisingly, these 27 DEL showed down-regulation in Taichung 65-4x compared to Taichung 65 (Additional file 1: Fig. S7A). In addition, 25 (DEL-anther: type 2) and 40 DEL (DEL-anther: type 3) were overlapped with the anther-preferred-2x and anther-preferred-4x lncRNAs, respectively. Similarly, 16 DEL were found to be associated with co-ovary-preferred lncRNAs (DEL-ovary: type 1). Interestingly, 15 out of 16 DEL were found to be down-regulated in Taichung 65-4x compared to Taichung 65, except TCONS_00019924 (Additional file 1: Fig. S7B). Six (DEL-ovary: type 2) and three DEL (DEL-ovary: type 3) were found to be overlapped with the ovary-preferred-2x and ovary-preferred-4x lncRNAs, respectively. The rest of the DEL in anther/ovary was defined as type 4.

In addition, we used BLAST search to compare the sequences and 160 lncRNAs displayed more than 90% sequence similarity with Zhang et al. [27]. Of these, 56 showed differential expression patterns between Taichung 65-4x and Taichung 65 (Additional file 2: Table S4). Among the 56 DEL, 18 showed differential expressions in Taichung 65-4x anthers, which highly expressed in anthers [27], such as TCONS_00130461. Two DEL were found to be differentially expressed in Taichung 65-4x ovaries that showed pistils expression patterns in Zhang’s results, such as TCONS_00054234.

### Small RNA populations and their relationships with differentially expressed lncRNAs in autotetraploid rice during meiosis

By small RNAs sequencing, 1034 miRNAs (i.e. 357 known miRNAs and 677 novel miRNAs) and 17,609 phasiRNAs (i.e. 15,992 were 21 nt in length and 1617 were 24 nt) were detected in the rice reproductive tissues (Additional file 2: Table S5 and S6). According to the model described by Boerner and McGinnis [29], these types of small RNAs were aligned to the 4859 confident lncRNAs to detect the small RNAs precursor in lncRNAs.

By aligning miRNA precursors to lncRNAs, 27 lncRNAs were predicted to be the precursors of 43 miRNAs (Additional file 2: Table S7). Nine of these showed differential expression patterns in autotetraploid rice anther, and one DEL-anther (Type 1) was corresponding to the differentially expressed miRNA (DEM) (i.e. TCONS_00160902 was predicted as a precursor of *zma-miR2275b-5p_1ss12CG*). A total of 98 lncRNAs were predicted to be the precursors of 949 phasiRNAs, including 63 lncRNAs as precursors to 433 21 nt-phasiRNAs (2.70%) and 35 lncRNAs as precursors to 516 24 nt-phasiRNAs (31.91%). Of these 98 lncRNAs, 29 showed differential expression patterns (down-regulation) in autotetraploid rice anther compared to diploid, but no one found to be differentially expressed in ovary of autotetraploid rice (Additional file 2: Table S8). Moreover, DEL-anther (Type 1) could generate most of phasiRNAs (270 phasiRNAs/13 DEL-anther) (Table 1). Interestingly, majority of the DEL (22 of 29 DEL) were found to be the precursors of 24 nt-phasiRNAs. In addition, nine down-regulated DEL were associated with 11 down-regulated phasiRNAs in Taichung 65-4x. Taken together, 39 DEL were associated with small RNAs, which could serve as precursors and showed down-regulation in autotetraploid rice (Additional file 2: Table S3).

**Table 1:** The number of small RNAs generated by differentially expressed lncRNAs in autotetraploid rice

Tissue	Small RNAs	Type 1		Type 2		Type 3		Type 4	
		No. DEL	No. small RNAs	No. DEL	No. small RNAs	No. DEL	No. small RNAs	No. DEL	No. small RNAs
Anther	miRNAs	2	5	0	0	0	0	7	22
	phasiRNAs	13	270	10	152	1	20	5	25
Ovary	miRNAs	2	8	0	0	0	0	0	0
	phasiRNAs	0	0	0	0	0	0	0	0

Type 1: The DEL overlapped with the co-anther-preferred/co-ovary-preferred lncRNAs of Taichung 65-4x and Taichung 65

Type 2: The DEL overlapped with anther-preferred/ovary-preferred lncRNAs of Taichung 65

Type 3: The DEL overlapped with anther-preferred/ovary-preferred lncRNAs of Taichung 65-4x

Type 4: The rest of the DEL in anther/ovary

Surprisingly, we could not identify any DEL-anther/ovary that predicted to be the target of a DEM in autotetraploid rice during meiosis by psRNATarget [31] (Additional file 2: Table S9). Moreover, there was no DEL-anther or DEL-ovary that could be the target mimic of the DEM in Taichung 65-4x, which decoy miRNAs and blocked the interaction between miRNAs and their authentic targets.

### Repetitive elements were associated with differentially expressed lncRNAs in autotetraploid rice during meiosis

**Fig. 3:**
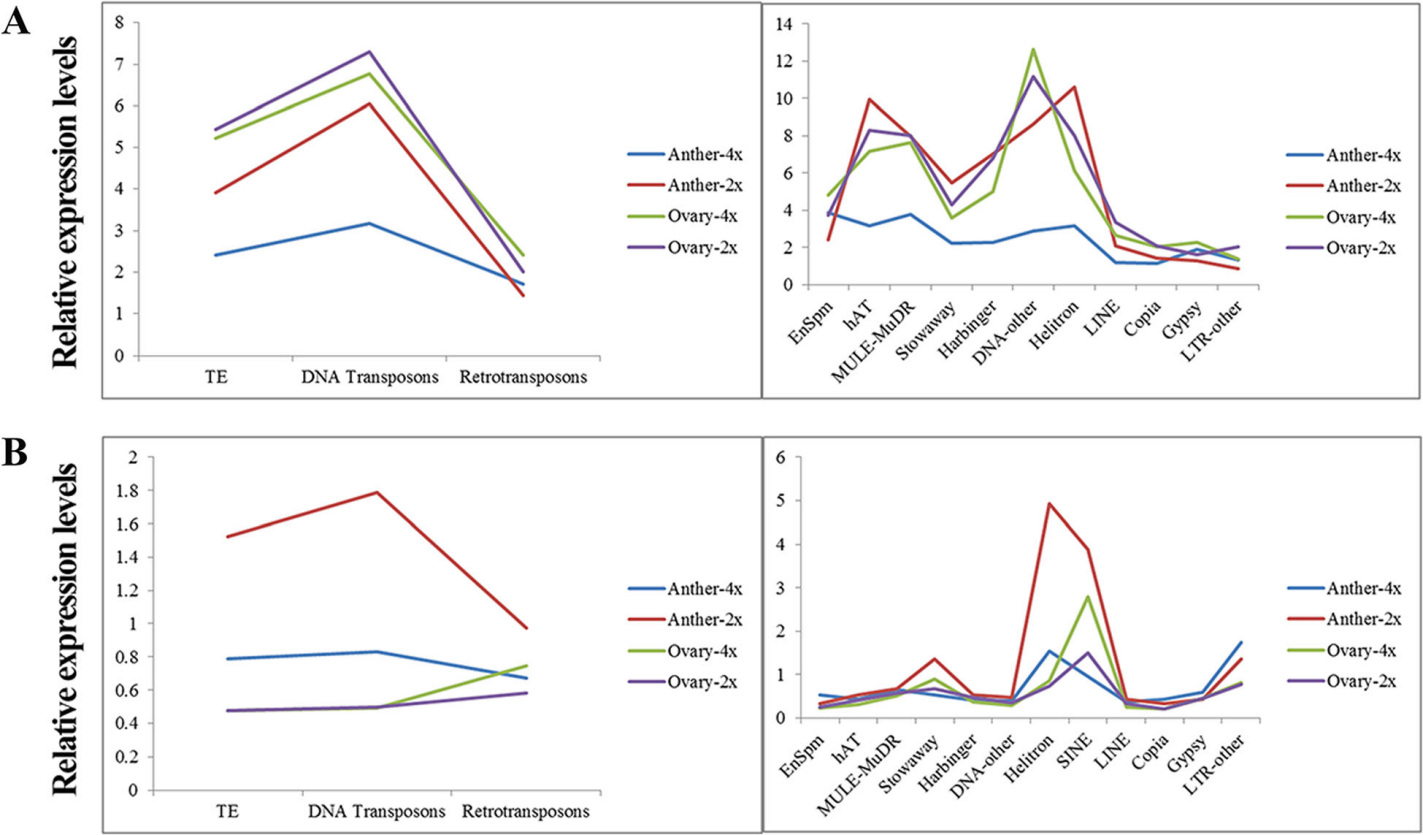
The relative expression levels of transposable elements (TE)-associated genes (**a**) and lncRNAs (**b**) in autotetraploid and diploid rice

A series of transposable elements (TEs) genes in anther and ovary were observed, and the relative expression levels of TEs were more abundant in the anthers of Taichung 65 than Taichung 65-4x, especially DNA transposons (i.e. Class II TEs, such as the Helitron family) (Fig. 3A). However, no remarkable changes in TEs were observed in ovary between diploid and autotetraploid rice. Majority of the lncRNAs (73.22%) were overlapped with repeat sequences. Of these TEs-associated lncRNAs, 66.36% carried Class II and 19.08% carried retrotransposons (Class I), while the remaining 14.56% contained both of them. Helitron family was the most frequent sub-class in Class II, whereas the Gypsy family constituted a major portion of Class I (Additional file 1: Fig. S8). The relative expression levels of TEs-lncRNAs also showed similar distribution of TEs genes between Taichung 65-4x and Taichung 65 (Fig. 3B). The differential expression patterns of some TEs and TEs-lncRNAs in anther were confirmed by qRT-PCR (Additional file 1: Fig. S9).

The TEs-associated lncRNAs showed differential expressions in autotetraploid rice anther and ovary, with a percentage of 73.78% (318/431) and 81.25% (26/32) in DEL, respectively (Additional file 2: Table S10). Of the TEs-associated DEL (TEs-DEL), down-regulated TEs-DEL-anther and TEs-DEL-ovary were mostly found to be associated with TEs (Table 2). Down-regulated TEs-DEL-anther were abundantly found in Class II, except EnSpm family, whereas up-regulated TEs-DEL-anther have a great relationship with Gypsy families of Class I in Taichung 65-4x anther. In addition, Helitron and Stowaway families were more related to TEs-DEL-anther (Type 1) compared to other types, and Helitron family was also detected in TEs-DEL-ovary (Type 1) (Additional file 3: Table S11).

**Table 2:** Distribution of the transposable elements (TEs)-related differentially expressed lncRNAs (DEL) in autotetraploid rice

Transposable elements	Family	Anther		Ovary	
		Down	up	Down	up
DNA Transposons	Helitron	50	4	8	1
	Harbinger	49	6	3	0
	Stowaway	47	1	8	4
	MULE-MuDR	38	11	2	1
	hAT	30	1	6	1
	DNA-other	26	1	2	0
	EnSpm	26	70	4	0
Retrotransposons	SINE	8	3	1	1
	LINE	12	0	1	0
	LTR-other	5	0	0	0
	Copia	6	10	0	0
	Gypsy	20	75	4	3

### Target prediction of differentially expressed lncRNAs and classification of pollen mother cell (PMC) and embryo sac mother cell (EMC) meiosis-related targets

To identify the potential targets of lncRNAs could help us to uncover the functions of lncRNAs. In total, 942 potential cis-regulated target genes and 3769 potential trans-regulated target genes were detected, and their target genes also showed differential expression patterns (hereafter referred as DEG) (Additional file 3: Table S12 and S13). Among the potential targets of the DEL-anther, 25 Gene Ontology (GO) terms were significantly categorized, including flower development, DNA binding and nucleus (Additional file 3: Table S14). Beside, only eight GO terms were significantly enriched among the DEL-ovary, such as response to abiotic stimulus.

Furthermore, we evaluated relationship between the targets predicted by DEM and DEL. A total of 104 and 108 predicted targets were identified from the DEM of anther (DEM-anther) and ovary (DEM-ovary) in Taichung 65-4x compared to the diploid rice, respectively (Additional file 3: Table S15). 76 and 81 targets were specifically predicted by DEM-anther and DEM-ovary, whereas 3739 and 328 targets were specifically predicted by DEL-anther and DEL-ovary, respectively (Additional file 1: Fig. S10). GO analysis showed that plastid was specifically enriched in the targets of DEM-anther, while anatomical structure morphogenesis, regulation of cell size and cell differentiation were specifically detected in the targets of DEM-ovary. Moreover, nucleus, response to endogenous stimulus and nucleic acid binding were specifically found in the targets of DEL-anther, and response to abiotic stimulus, secondary metabolic process, and response to extracellular stimulus were specifically identified in the targets of DEL-ovary (Additional file 3: Table S16).

Moreover, we compared the targets with the previous studies to identify the most important meiosis-related targets [32–35]. By using the predicted targets of DEL-anther, 237 DEL-anther were found to be associated with 110 meiosis-related genes (DEL-PMC) (Additional file 3: Table S17). Of these targets, six rice meiosis genes, including *LOC_Os01g39630* [36] (RAD51C, annotated as DNA repair protein Rad51) predicted by TCONS_00049558, *LOC_Os03g58600* [37] (MEL1, encoded PAZ domain containing protein) predicted by TCONS_00068868, and *LOC_Os01g68870* [38] (OsMSP1, annotated as leucine-rich repeat receptor protein kinase EXS precursor), *LOC_Os02g57270* [39] (OsTBP1, annotated as MYB family transcription factor), *LOC_Os08g16610* [40] (OsRad21–3, annotated as Rad21/Rec8 like protein), and *LOC_Os12g31370* [41] (RAD51A2, annotated as DNA repair protein Rad51) predicted by TCONS_00131142, related to the male meiocytes were identified and showed down-regulation in the present study. Thirteen putative meiosis-related genes displayed down-regulation in autotetraploid rice anther, such as *LOC_Os03g12730* [42] (the homologous *Arabidopsis* gene is BAM1, annotated as receptor protein kinase CLAVATA1 precursor) predicted by TCONS_00068868, *LOC_Os04g30790* [43] (the homologous *Arabidopsis* gene is XRI1, annotated as expressed protein) and *LOC_Os11g12620* [42] (the homologous *Arabidopsis* gene is BAM1, annotated as receptor protein kinase CLAVATA1 precursor) predicted by TCONS_00055980, *LOC_Os12g34510* [44] (the homologous *Arabidopsis* gene is H2AX, encoded Core histone H2A/H2B/H3/H4 domain containing protein) predicted by TCONS_00068868 and TCONS_00121884, and *LOC_Os12g41350* [44] (the homologous *Arabidopsis* gene is ASY1, annotated as meiotic asynaptic mutant 1) predicted by TCONS_00057811. In addition, 13 targets showed meiosis stage-specific gene expressions [32, 33], and 81 targets exhibited differential expression patterns in autotetraploid compared to the diploid rice during meiosis [11]. Of these 81 targets, 28 targets exhibited regulation patterns similar to Wu et al. [11], such as *LOC_Os03g11540* (RPA1B - Putative single-stranded DNA binding complex subunit 1) and *LOC_Os05g14590* (MCM6 - Putative minichromosome maintenance MCM complex subunit 6) regulated by TCONS_00055980 and TCONS_00130461, respectively.

Of the targets of DEL-ovary, 20 DEL showed relationship to the meiosis stage (DEL-EMC) (Additional file 3: Table S18), and 39 targets showed EMC specific expressions [34, 35]. Six genes, such as *LOC_Os06g12740* (expressed protein) predicted by TCONS_00121908 and *LOC_Os10g32740* (zinc finger family protein) predicted by TCONS_00114760, were enriched in zygotene and pachytene of EMC [34]. 28 genes showed the highest expressions in late meiosis stage of megasporocyte [34], such as *LOC_Os12g39420* (nucleobase-ascorbate transporter), *LOC_Os06g44300* (WAX2), *LOC_Os08g45170* (carboxyl-terminal peptidase) and *LOC_Os05g06460* (dihydrolipoyl dehydrogenase) predicted by TCONS_00144592, TCONS_00037710, TCONS_00121908 and TCONS_00019924, respectively. Interestingly, we found that PMC and EMC meiosis-related DEG were absolutely different, and most of them displayed down-regulation in autotetraploid rice PMC (106/110) and EMC (30/39).

Additionally, 14 meiosis-related DEG displayed protein-protein interactions (PPI) (with 8 and 6 DEG belong to two sub-networks) in EMC, including *LOC_Os05g06460* (targeted by TCONS_00019924) interacted with *LOC_Os03g18740* (oxidoreductase, short chain dehydrogenase/reductase family, targeted by TCONS_00114760), and *LOC_Os03g15960* (connectivity = 3) annotated as hsp20/alpha crystallin family protein and targeted by TCONS_00144592 (Additional file 1: Fig. S11). A huge PPI network of 47 meiosis-related DEG, predicted by DEL-PMC, was found in PMC. Two sub-networks were established, including one contained two DEG, and the remaining DEG belonged to second sub-network, which was the main network. Two meiosis DEG, *LOC_Os11g12620* (receptor protein kinase CLAVATA1 precursor, regulated by TCONS_00055980) and *LOC_Os12g41350* (meiotic asynaptic mutant 1, predicted by TCONS_00057811), as well as *LOC_Os01g39630* (RAD51C), were found in this huge sub-network (Additional file 1: Fig. S12).

**Fig. 4:**
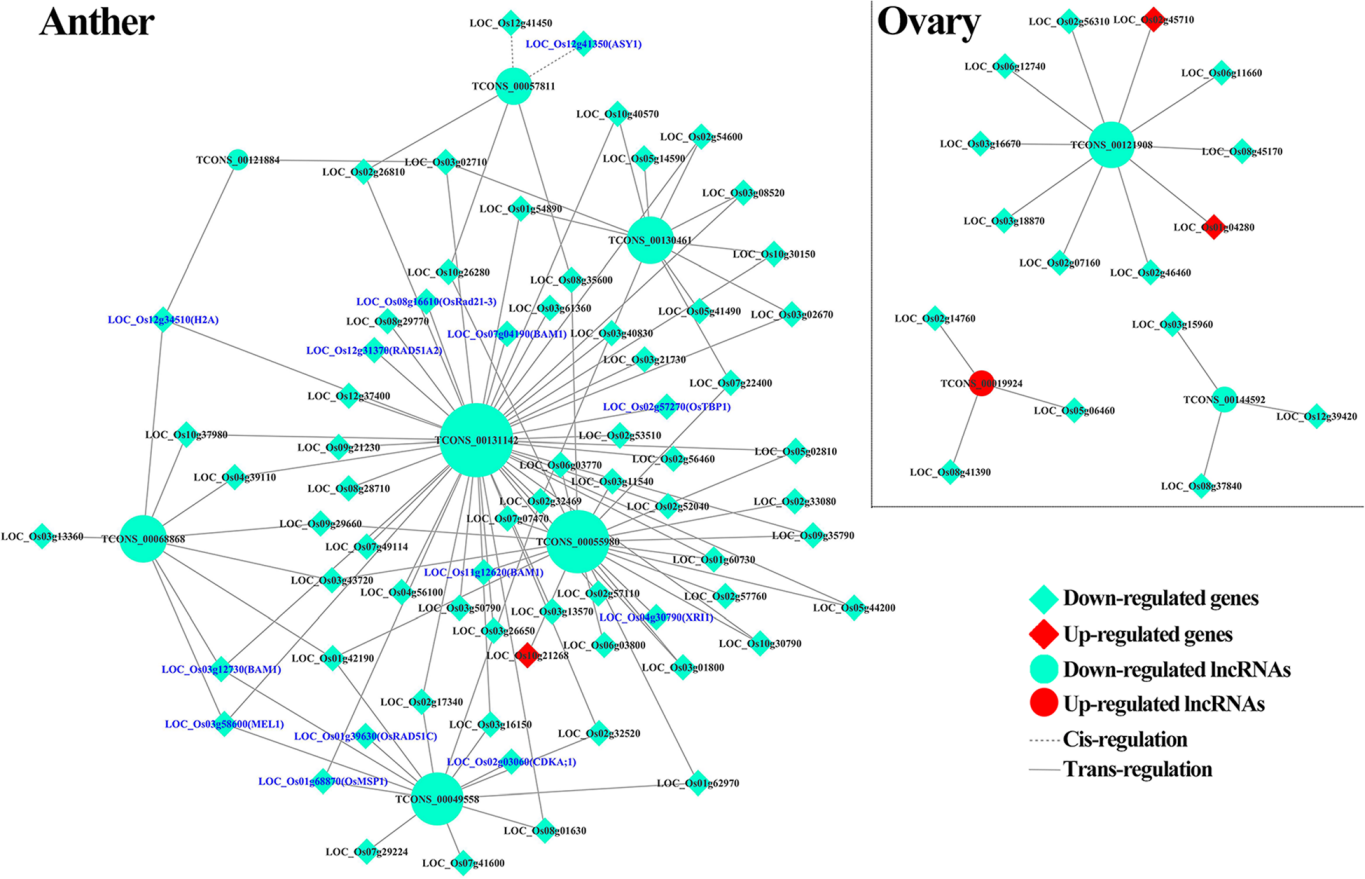
Prediction of some meiosis-related lncRNA-mRNA association network. The lncRNAs and the target genes showed differential expression patterns and were related to the meiosis. Blue front represents the meiosis genes

We categorized the important DELs associated with meiosis-related DEG as shown in Fig. 4, Table 3 and Table 4. The regulatory networks between DELs and DEG may important during anther and ovary development. To confirm the differential expression levels of lncRNAs and target genes, 23 DEL, including 21 DEL-Type 1 and 26 meiosis-related genes were selected to validate the expression patterns using qRT-PCR. The qRT-PCR generally confirmed the high-throughput sequencing results, proving the reliability of RNA-sequencing data used in the present study (Additional file 1: Fig. S13 and S14).

**Table 3:** List of PMC meiosis-related differentially expressed lncRNAs in the autotetraploid rice

DEL-PMC	Regulation	Targets	Annotation	Regulation	small RNAs precursors	Transposable elements	Reference
TCONS_00026633	down	*LOC_Os08g01630*	DNA repair metallo-beta-lactamase, putative, expressed	down		Class I and Class II	Wu et al. [11]
TCONS_00049558	down	*LOC_Os01g39630*	DNA repair protein Rad51, putative, expressed	down	TCONS_00049558(*)		Kou et al. [36] (RAD51C)
TCONS_00055980	down	*LOC_Os04g30790*	expressed protein	down			Dean et al. [43] (XRI1)
	down	*LOC_Os11g12620*	receptor protein kinase CLAVATA1 precursor, putative, expressed	down			Hord et al. [42] (BAM1)
	down	*LOC_Os03g11540*	RPA1B - Putative single-stranded DNA binding complex subunit 1, expressed	down			Wu et al. [11]
TCONS_00057811	down	*LOC_Os12g41350*	meiotic asynaptic mutant 1, putative, expressed	down			Sanchez-Moran et al. [44] (ASY1)
	down	*LOC_Os12g41450*	F-box domain containing protein, expressed	down			Deveshwar et al. [33]
TCONS_00068868	down	*LOC_Os03g58600*	PAZ domain containing protein, putative, expressed	down	TCONS_00068868(*)	Class II	Nonomura et al. [37] (MEL1)
	down	*LOC_Os03g12730*	receptor protein kinase CLAVATA1 precursor, putative, expressed	down		Class II	Hord et al. [42] (BAM1)
	down	*LOC_Os12g34510*	Core histone H2A/H2B/H3/H4 domain containing protein, putative, expressed	down		Class II	Sanchez-Moran et al. [44] (H2AX)
TCONS_00091337	down	*LOC_Os01g60730*	RING-H2 finger protein, putative, expressed	down		Class I and Class II	Wu et al. [11]
TCONS_00121645	down	*LOC_Os02g57760*	O-methyltransferase, putative, expressed	down	TCONS_00121645(*)	Class I and Class II	Fujita et al. [32]
TCONS_00121884	down	*LOC_Os12g34510*	Core histone H2A/H2B/H3/H4 domain containing protein, putative, expressed	down	TCONS_00121884(*)	Class I and Class II	Sanchez-Moran et al. [44] (H2AX)
TCONS_00130461	down	*LOC_Os05g14590*	MCM6 - Putative minichromosome maintenance MCM complex subunit 6, expressed	down	TCONS_00130461(*)	Class I and Class II	Wu et al. [11]
TCONS_00131142	down	*LOC_Os01g68870*	leucine-rich repeat receptor protein kinase EXS precursor, putative, expressed	down	TCONS_00131142	Class II	Nonomura et al. [38] (OsMSP1)
	down	*LOC_Os02g57270*	MYB family transcription factor, putative, expressed	down		Class II	Hong et al. [39] (OsTBP1)
	down	*LOC_Os08g16610*	Rad21/Rec8 like protein, putative, expressed	down		Class II	Tao et al. [40] (OsRad21–3)
	down	*LOC_Os12g31370*	DNA repair protein Rad51, putative, expressed	down		Class II	Morozumi et al. [41] (RAD51A2)
TCONS_00136741	down	*LOC_Os04g43300*	BRCA1 C Terminus domain containing protein, expressed	down		Class II	Wu et al. [11]

Down: down-regulated in autotetraploid rice

Small RNAs precursors: DEL serves as miRNAs/phasiRNAs precursors

Class I: Retrotransposons; Class II: DNA Transposons

(*): the small RNAs showed differential expression in autotetraploid rice

**Table 4:** List of EMC meiosis-related differentially expressed lncRNAs in the autotetraploid rice

DEL-EMC	Regulation	Targets	Annotation	Regulation	small RNAs precursors	Transposable elements	Reference
TCONS_00019924	up	*LOC_Os05g06460*	dihydrolipoyl dehydrogenase, putative, expressed	down		Class I and Class II	Kubo et al. [34]
TCONS_00023646	down	*LOC_Os01g67850*	zinc finger, RING-type, putative, expressed	down		Class I and Class II	Wu et al. [35]
TCONS_00037710	down	*LOC_Os06g44300*	WAX2, putative, expressed	down		Class II	Kubo et al. [34]
TCONS_00115107	down	*LOC_Os01g08560*	DnaK family protein, putative, expressed	down		Class II	Kubo et al. [34]
TCONS_00121908	down	*LOC_Os02g46460*	peptide transporter PTR2, putative, expressed	down		Class II	Wu et al. [35]
	down	*LOC_Os06g12740*	expressed protein	down		Class II	Kubo et al. [34]
	down	*LOC_Os08g45170*	carboxyl-terminal peptidase, putative, expressed	down		Class II	Kubo et al. [34]
TCONS_00134306	down	*LOC_Os02g41630*	phenylalanine ammonia-lyase, putative, expressed	down		Class I and Class II	Kubo et al. [34]
TCONS_00144592	down	*LOC_Os03g15960*	hsp20/alpha crystallin family protein, putative, expressed	down		Class II	Kubo et al. [34]
	down	*LOC_Os12g39420*	nucleobase-ascorbate transporter, putative, expressed	down		Class II	Kubo et al. [34]

Up: up-regulated in autotetraploid rice

Down: down-regulated in autotetraploid rice

Small RNAs precursors: DEL serves as miRNAs/phasiRNAs precursors

Class I: Retrotransposons; Class II: DNA Transposons

### Overexpression of lncRNA57811 reduced rice seed set

**Fig. 5:**
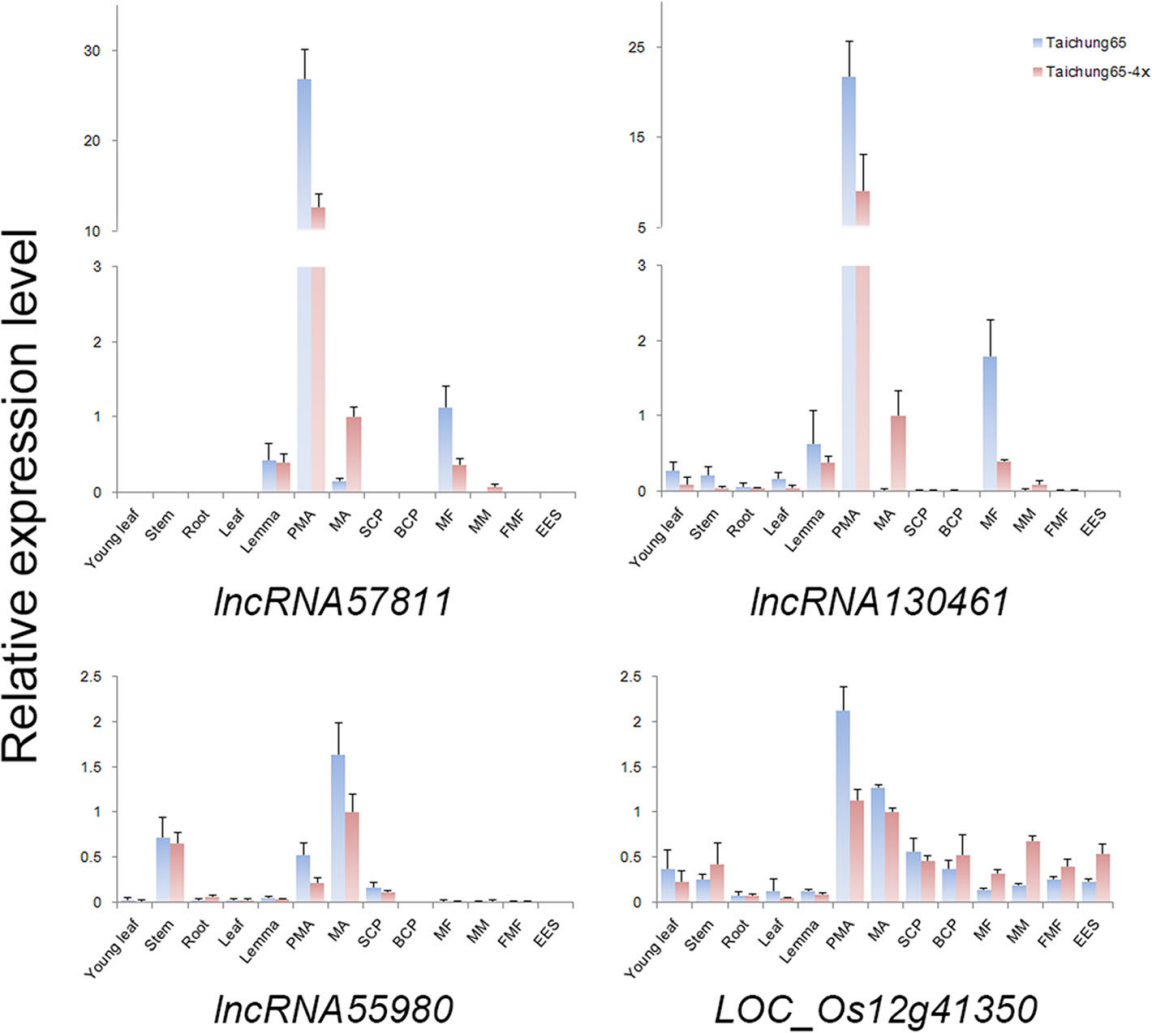
The expression patterns of lncRNAs in Taichung 65-4x and Taichung 65. PMA: Premeiotic interphase to meiosis prophase I, MA: Metaphase I to meiosis II, SCP: Single microspore developing-stage, BCP: Bi-cellular pollen stage to mature pollen, MF: Megasporocyte formation stage to meiosis prophase I, MM: Megasporocyte metaphase I to meiosis II, FMF: Functional megaspore formation to embryo sac mitotic stage, EES: Eight-nucleate embryo sac developing-stage to mature embryo sac. The stage of ovary was corresponding to the stage of anther. Error bars represent the standard deviation (SD) of three biological replicates

**Fig. 6:**
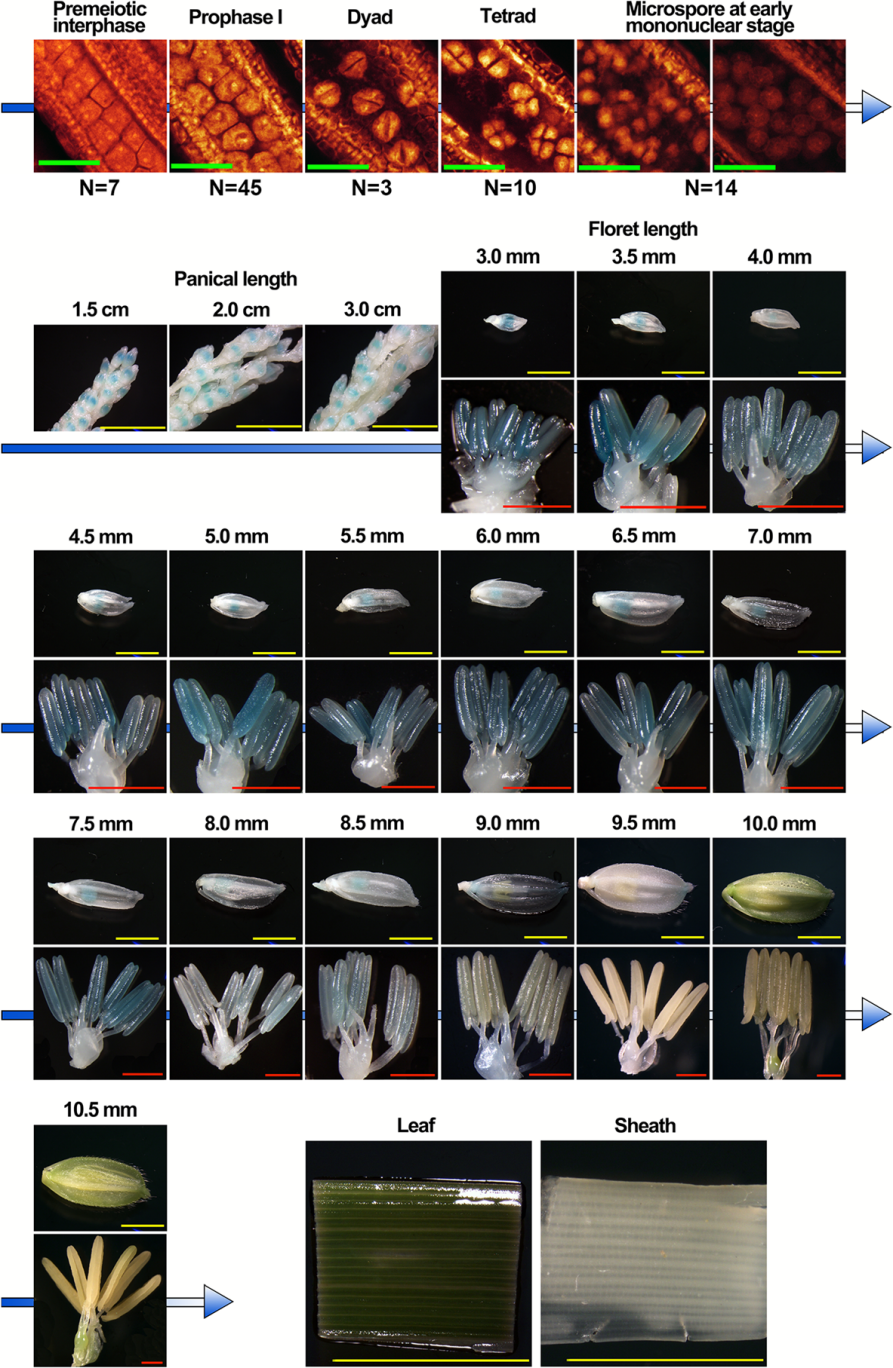
GUS staining assay of lncRNA57811-Pro::GUS. ‘N’ indicates the number of the GUS-stained anthers observed at particular development stage by WE-CLSM. Green Bars = 40 μm. The stained tissues were photographed using LEICA MZ16. Yellow Bars = 4 mm and Red Bars = 1 mm

To test the potential functions of the DELs, TCONS_00057811 (lncRNA57811), TCONS_00130461 (lncRNA130461) and TCONS_00055980 (lncRNA55980) were selected for further analysis, which showed anther-specific expression patterns. Of these, lncRNA57811 and lncRNA130461 were highly expressed from pre-meiotic interphase to prophase I during pollen development, whereas lncRNA55980 was highly expressed during late meiosis stage (Fig. 5). Additionally, lncRNA57811 and lncRNA130461 exhibited down-regulation at early meiosis stage, while significantly up-regulated at later meiosis stage in Taichung 65-4x compared to diploid counterpart. Then, the expression patterns of lncRNA57811 were confirmed by GUS stained assays (Fig. 6), and GUS activity was visualized only in anther at early pollen development stage i.e. from pre-meiotic stage to prophase I. The staining intensity decreased dramatically after single microspore stage.

**Fig. 7:**
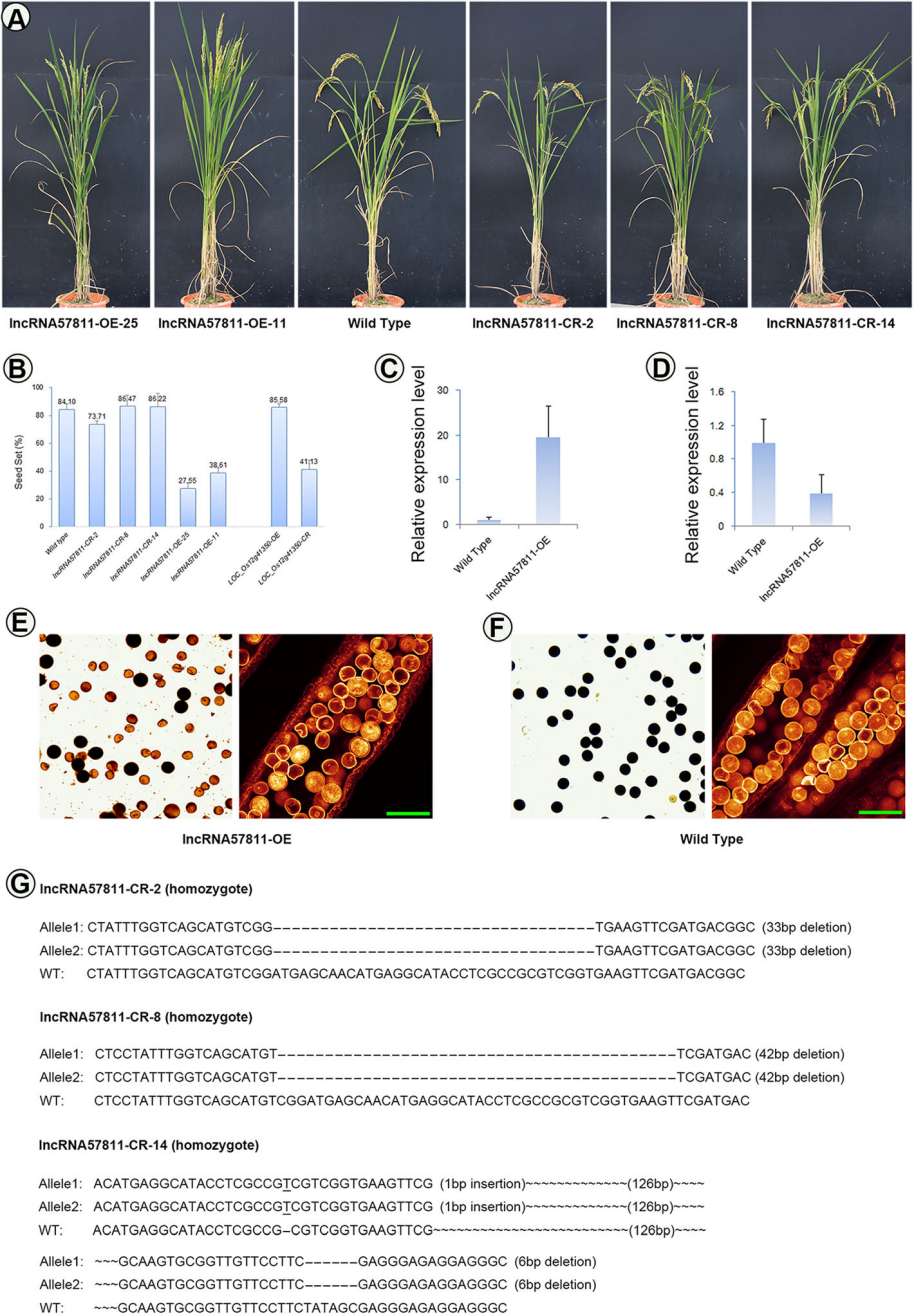
Phenotype of lncRNA57811-CR and lncRNA57811-OE in T1 generation. **a** Comparison between the wild-type plant and lncRNA57811 mutant plant after heading. **b** The seed set of Taichung 65, lncRNA57811-CR and lncRNA57811-OE lines. **c** lncRNA57811-OE mutants have high expression levels of lncRNA57811 compared to wild type. **d** *LOC_Os12g41350* showed down-regulation in lncRNA57811-OE mutants. **e**, **f** lncRNA57811-OE mutants displayed lower pollen fertility than the wild type. **g** Genotype of lncRNA57811-CR lines

lncRNA57811, lncRNA130461 and lncRNA55980 mutants with the background of Taichung 65 were generated by using CRISPR/Cas9 technology; however, CRISPR/Cas9 editing did not show obvious seed phenotypes in T0 generation. We further focused on the lncRNA57811 by generating its overexpressed mutants, and then planted T1 generation. The seed set of the independent CRISPR lines of lncRNA57811 in T1 generation was similar to the wild type, even though 42 bases deletion was detected on its gene body (Fig. 6, Additional file 1: Fig. S15). Interestingly, overexpression of lncRNA57811 (57811OE) showed distinctly low seed set compared to wild type in T1 generation (Fig. 7). The seed set of 57811OE-11 and 57811OE-25 lines were 38.61 and 27.55%, respectively. The pollen fertility of 57811OE was 29.70%, which was significantly lower than the wild type (95.75%). Furthermore, overexpression and CRISPR mutants of *LOC_Os12g41350* (*12 g*) were obtained. *12 g* was preferentially expressed in reproductive tissues, while poorly expressed in vegetative tissues. Overexpression of *12 g* showed similar morphology to wild type (Additional file 1: Fig. S16). One homozygous mutant with an 8 bp deletion, which resulted in premature termination of the protein sequence, was identified in T0 generation. This line displayed semi-sterility with a seed set of 41.13% in T1 generation. Moreover, *LOC_Os12g41350* showed down-regulation in 57811OE mutants.

## Discussion

### LncRNAs associated with rice reproductive development shared common characteristics with other species

More than 50% of lncRNAs showed tissue specific patterns in rice anthers and strawberry pollens, which revealed that the expression patterns of lncRNAs play important role in reproductive tissues [24, 27]. In the present study, we characterized 4859 reliable lncRNAs during meiotic stages of anther and ovary in autotetraploid and diploid rice. These lncRNAs shared similar features with the other mammals and plant species, such as shorter transcript length and lower expression levels than protein-coding genes [45–48]. On the other hand, only a few rice reproductive lncRNAs with conserved sequences among the other plant species were detected, which indicated that rice reproductive lncRNAs may undergo a rapid evolvement [47, 49]. However, 11 lncRNAs were found to be conserved in *Arabidopsis*, *Brachypodium*, *Zea mays* and *Sorghum bicolor,* which might be the housekeeping lncRNAs and carry out important functions in angiosperms. Moreover, full-length of 10 lncRNAs were obtained by RACE, and six of them were found to be conserved among the neo-tetraploid, autotetraploid, diploid and wild rice, especially lncRNA57811, which indicated that these reproductive-related lncRNAs may play important functions.

### LncRNAs act as small RNAs precursors in autotetraploid rice that might play potential role in PMC and EMC meiosis

The majority of lncRNAs act as precursors for small RNA, and siRNAs seem to be the major small RNA population and many lncRNAs serve as precursors for siRNAs [23, 25, 29]. According to a previous study about *Arabidopsis*, the siRNAs are generated by Pol IV, and their precursors are 30–40 nt [50], which is in contrast to the model that lncRNAs could be defined as the transcripts longer than 200 bp [22]. So, we focused on the miRNAs and phasiRNAs, and 37 DEL served as precursors for small RNA and all of them displayed down-regulation in autotetraploid rice, which suggested that the down-regulated DEL were associated with small RNAs generation in autotetraploid rice, particularly with anther. PhasiRNAs were found to be associated with male meiocytes in rice and maize [51, 52]. Another study revealed that a SNP in PMS1T (lncRNA), nearby *miR2118* recognition site, lead to differential accumulation of the 21 nt-phasiRNAs and resulted in male sterility under long-day conditions [53]. Recently, down-regulation of 24 nt-phasiRNAs was consistent with *osa-miR2275d* during pollen development in autotetraploid rice [20]. Our results demonstrated that down-regulated DEL, especially DEL-PMC (Type 1), may cause low expression of phasiRNAs, which could harm meiocytes of autotetraploid rice. LncRNAs were predicted to be miRNAs targets or target mimics in rice and maize [54], however, these results are hard to detect in autotetraploid rice. We speculated that lncRNAs prefer to serve as the small RNA precursors rather than the miRNAs targets or decoys in rice, even in the autotetraploid rice. Moreover, different kinds of predicted targets between DEL and DEM were detected and only few targets (23) were co-regulated by DEL and DEM in our study. These specific targets showed different categories of GO functions in autotetraploid rice anther and ovary, which suggested that lncRNAs and miRNAs take part in various functions during meiosis in autotetraploid rice.

### Low expression levels of transposable elements, associated with lncRNAs, may cause great harm to male and female meiocytes in autotetraploid rice

WGD events are an obvious route to genome expansion, and the increase genome size owing to the rapid proliferation of transposable elements [55]. Over 70% rice reproductive lncRNAs were associated with TEs in the present study, and these results were consistent with other plant species [25, 56]. Zhang et al. [57] indicated that hypermethylation of class II DNA transposons may suppress the expression of neighboring genes, which become a “genome shock” to adapt the genome-dosage effects in autotetraploid rice. In our previous studies, the differentially expressed siRNAs were found to be associated with TEs during male meiosis, which implied the relationship between TEs and sterility in autotetraploid rice [20, 21]. One interesting finding about *Arabidopsis* male meiocytes illustrated that TEs were abundant and specifically expressed in meiocytes [58, 59]. The activated TEs only detected in vegetative cells but not in sperm cells of the haploid pollen [60]. Male meiocytes represent the final stage of the diploid phase with reactivated TEs, which may involve in chromatin structures and facilitated the precession of meiosis [58, 59]. TEs-lncRNAs detected in male sunflower meiocytes were found to be associated with the different recombination rates between domesticated and wild sunflower [25]. Another study reported that the expression levels of TE-lncRNAs were the highest in testis [61], and important in the biology of normal testis [62]. We observed low expression levels of TEs and TEs-lncRNAs, and down-regulation of TEs-DEL (Helitron and Stowaway families of Class II) in autotetraploid rice may disturb the chromatin structures or have harmful effects on meiocytes. The TEs and TEs-lncRNAs associated with meiocytes required further studies.

### Meiosis-related lncRNAs may play key roles during PMC and EMC meiosis in autotetraploid rice

LncRNAs are revealed to execute direct meiotic roles during meiosis [63, 64], such as UPGRADE2, which was highly up-regulated and attained neofunctionalization in the context of apomeiosis for pollen development in *Boechera* species [65], and LDMAR, a SNP could repress the expression level of LDMAR and cause male sterility in rice [26]. Here, 237 DEL-PMC and 20 DEL-EMC were found to be associated with 110 and 39 meiosis-related genes. Most of these meiosis-related genes showed down-regulation and have protein-protein interactions in autotetraploid rice that related to the abnormal meiosis and sterility in our previous study [8, 11], such as *LOC_Os01g39630* (RAD51C) and *LOC_Os02g57270* (OsTBP1), which is required for the meiosis of male gametocytes [36, 39]. We detected TCONS_00068868, the DEL-PMC (Type 1), which showed down-regulation during meiosis of autotetraploid rice anther, and its target *LOC_Os03g58600* (MEL1) showed the same differential expression pattern and validated by qRT-PCR. Rice MEL1 is a key gene involved in meiosis that regulates the cell division of pre-meiotic germ cells, and the proper modification of meiotic chromosomes [37, 66]. Moreover, MEL1 was preferentially associated with 21 nt-phasiRNAs, which play important role during meiosis [51]. We speculated that the abnormal expression of MEL1 might cause abnormal meiosis behavior in autotetraploid rice, and the down-regulation of its lncRNA regulators may triggered the abnormal expressions.

In addition, we detected ten DEL-EMC (Type 1) that showed great relationships with the 27 embryo sac meiosis-related genes [34, 35]. Though the functions of these targets remain largely unknown, they highly expressed and showed meiosis specific expressions during megasporocytes, which suggested their key roles during the meiosis procession of megasporocytes. The regulation of EMC meiosis-related targets in autotetraploid rice was confirmed by the qRT-PCR, such as *LOC_Os12g39420*, *LOC_Os02g46460* and *LOC_Os05g06460* regulated by TCONS_00144592, TCONS_00121908 and TCONS_00019924, respectively. These lncRNAs/target pairs probably played essential roles and involved in female meiocytes development of autotetraploid rice, and their abnormal expression patterns might have a strong effect on the embryo sac fertility of autotetraploid rice. Overall, the list of meiosis-DELs in rice anther/ovary will be beneficial for future studies in plant reproduction.

Overexpression of lncRNA57811 showed low seed set and pollen sterility in T1 generation, while CRISPR/Cas9 editing of lncRNA57811 displayed similar morphology when compared to Taichung 65. lncRNA57811 showed anther specific pattern and highly expressed from pre-meiotic interphase to prophase I during pollen development, which demonstrated that lncRNA57811 play key role at early meiosis stage. The higher expression levels of lncRNA57811 were detected in Taichung 65-4x and Taichung 65 at pre meiosis, however, significantly higher expression level of lncRNA57811 was detected in Taichung 65-4x than Taichung 65 during late meiosis stage. These results indicated that the irregular expression pattern of lncRNA57811 might be the main reason for low fertility in Taichung 65-4x.

For knock-down analysis of lncRNAs, CRISPR/Cas9 editing system should not be a first choice tool. In this study, three lncRNAs were selected for generating the editing-mutant by CRISPR/Cas9; however, no obvious change was observed in morphology. This might be happened because 1) CRISPR/Cas9 usually causes frame shift and base replacement in protein sequences, but lncRNAs are the noncoding RNA and no protein. 2) lncRNAs are full of repetitive sequences, deletions/insertions mutation in one repetitive region, and their functions might be replaced by other repetitive regions. 3) the key regions of lncRNAs have not been be edited successfully so far. 4) the functions of these three lncRNAs could be replaced by other lncRNAs/genes. Recently, Wang et al. [28] used RNAi method to generate the knock-down mutants of lncRNA and acquired desired results. Therefore, there is a need to develop more methods to generate knock-down of lncRNAs in future. Otherwise, more specific-sgRNA targets of CRISPR/Cas9 editing system should be utilized to make sure about the whole/most length of lncRNA to be knocked-out.

*LOC_Os12g41350* (meiotic asynaptic mutant 1) was the predicted cis-target of lncRNA57811, and down-regulation of *LOC_Os12g41350* in Taichung 65-4x was confirmed by qRT-PCR. CRISPR/Cas9 editing of *LOC_Os12g41350* showed semi-sterility in T1 generation. Additionally, *LOC_Os12g41350* showed down-regulation in lncRNA57811-OE. The homologous gene of *LOC_Os12g41350*, namely *ASY1*, is an important protein required for synapsis and crossover formation during early meiosis in *Arabidopsis* [44]. Cytological studies have revealed that *ASY1* is localized to the regions of chromatin, associated with the axial/lateral elements of meiotic chromosomes, and required for morphogenesis of the synaptonemal complex [67]. Mutant lines *asy1* showed identical asynaptic phenotypes during male meiosis [68]. In addition, homologous asynapsis in meiotic prophase I could induce aneuploidy in autopolyploid *Arabidopsis asy1* [69]. Moreover, a single crossover per chromosome (reduce crossover rates) could prevent multivalent formation in autotetraploid plants [18]. We hypothesized that lncRNA57811 might be served as cis-regulatory element of the *LOC_Os12g41350* in rice, which resulted in pollen sterility. However, it required further experiments to confirm the relationship between lncRNA57811 and *LOC_Os12g41350*, and to know whether *LOC_Os12g41350* is a cis-target of lncRNA57811. The function and the regulation network of lncRNA57811 and *LOC_Os12g41350* during pollen development should be further analyzed.

## Conclusion

In this study, we utilized high-throughput sequencing to identify the long noncoding RNAs associated with meiosis in autotetraploid rice anther and ovary compared to diploid rice. A total of 4859 confident lncRNAs were identified, which shared common features with other species. Ten lncRNAs were validated by RACE assay, and six were found to be conversed in tetraploid rice. Of the DEL, 237 and 20 DEL were identified as PMC meiosis-related DEL and EMC meiosis-related DEL, respectively. Target prediction showed that these meiosis-related DEL were associated with some important meiosis genes. Interestingly, most of them were associated with the transposable elements and could act as small RNAs precursors, especially the anther/ovary-preferred lncRNAs in autotetraploid rice. Overexpression of lncRNA57811 showed low seed set and pollen sterility indicated that lncRNA played important function during pollen development in polyploid rice. Consequently, the significant differential expression profiles of long noncoding RNAs in the autotetraploid rice may play key roles in regulating the reproductive tissues, thus resulting in low fertility and poor seed set. Our findings provide a new insight on long noncoding RNA in autotetraploid rice reproduction.

## Methods

### Plant material

Autotetraploid rice, Taichung 65-4x, and its diploid rice, Taichung 65, were used in this study. Taichung 65 is a famous *japonica* cultivar from Taiwan and bred by Taichung agricultural improvement farm in 1929 [70]. Taichung 65 was kindly characterized and provided by Prof. Guiquan Zhang (College of Agriculture, South China Agricultural University), and is being used by our research group since 1989 [11, 71, 72]. Taichung 65 has been deposited in the International Rice Genebank Collection Information System (IRGCIS, http://www.irgcis.irri.org:81/grc/irgcishome.html) with the accession number of 9434 since 1962. Taichung 65-4x was developed from the chromosome doubling of Taichung 65 by colchicine and was self-crossed for more than 30 generations by our research group (Prof. Xiangdong Liu) at South China Agricultural University (Guangzhou, Guangdong, China). The voucher specimen of Taichung 65-4x has been deposited to our lab but not in any publicly available herbarium. These lines were planted at the experimental farm of South China Agricultural University under field conditions. Anthers at pollen development stages were collected from Taichung 65-4x and Taichung 65 according to Wu et al. [11]. The ovary tissues were collected from the same spikelet (corresponding anther). All the samples were stored at − 80 °C for RNA isolation. Three biological replications were used for each tissue for high-throughput RNA-sequencing.

### Transcriptome library construction and sequencing

Total RNA was extracted from the anthers and ovaries by using Trizol reagent (Invitrogen, CA, USA) following the manufacturer’s procedure. The quantity and purity of total RNA were analyzed by Bioanalyzer 2100 and RNA 6000 Nano LabChip Kit (Agilent, CA, USA) with RIN number > 7.0. Approximately 10 μg of total RNA was used to deplete ribosomal RNA by using Epicentre Ribo-Zero Gold Kit (Illumina, San Diego, USA). Divalent cations under high temperature were used to break poly(A)- or poly(A) + RNA fractions is into small pieces. The final cDNA library was constructed according to the methodology of mRNA-Seq sample preparation kit (Illumina, San Diego, USA). Finally, the paired-end sequencing was performed by Illumina Hiseq2500 (LC Sciences, Hangzhou, China) with an average insert size of 300 bp (±50 bp).

### Prediction and identification of lncRNAs

Prior to assembly, the low quality reads including reads containing sequencing adaptors, reads containing sequencing primer, nucleotide with quality score lower than 20 (Q < 20), were removed. After that, the clean reads were aligned to the *Oryza sativa* genome (ftp://ftp.jgi-psf.org/pub/compgen/phytozome/.v10.0/Osativa 204 v7.0/assembly/) using TopHat2 package [73]. The aligned reads were then used to assemble transcripts of each sample independently, using Cufflinks program [74]. The expression levels of the assembled transcripts were further calculated and normalized by Cufflinks using the measurement unit of fragments per kilobase of transcript per million fragments (FPKM). The prediction of lncRNAs from RNA-seq data was performed according to Sun et al. [75]. We discarded transcripts shorter than 200 bp, exons number less than one and transcripts with FPKM < 0.5. The transcripts with five class codes information (i.e. ‘i’, ‘j’, ‘u’, ‘o’, and ‘x’) were extracted. Class codes of ‘u’, ‘i’, and ‘x’ mean that the transcripts are in the intergenic, intronic, and antisense sequence, respectively, of a known gene. Class codes of ‘o’ and ‘j’ mean that the transcripts overlapped with known exons [74]. Then the coding potential of the remaining transcripts was evaluated using coding potential calculator (CPC) software [30] and Coding Noncoding Index (CNCI) software [75]. The transcripts with CPC score < = − 1 and CNCI score < = 0 were identified as the lncRNA candidates. LncRNAs with *P*-value < 0.05 and |log2 (fold change ratio)| > 1 were considered as differentially expressed lncRNAs.

### Small RNA library construction and sequencing

We further used about 1 μg of total RNA for the small RNA sequencing, and then prepare small RNA libraries according to protocol of TruSeq Small RNA Sample Prep Kits (Illumina, San Diego, USA). After that, we executed the single-end sequencing (36 bp) on an Illumina Hiseq2500 at the LC-BIO (Hangzhou, China) according to the manufacturer’s protocol. For the miRNAs analysis, the raw data was further processed with an in-house program, ACGT101-miR (LC Sciences, Houston, Texas, USA) [21]. First, the reads with common RNA families (snRNA, tRNA, snoRNA, rRNA), low complexity, repeats, junk and adapter dimers were removed. Then, the unique sequences (18-25 nt in length) were BLASTed to miRBase 20.0 (ftp://mirbase.org/pub/mirbase/) to identify known miRNAs. The novel miRNAs were predicted by using RNAfold software (http://rna.tbi.univie.ac.at/cgi-bin/RNAWebSuite/RNAfold.cgi). miRNAs with *P*-value < 0.05 and |log2 (fold change ratio)| > 1 were considered as differentially expressed miRNAs. Targeted genes of differentially expressed miRNAs were predicted by using TargetFinder [76]. For the phasiRNAs analysis, 21 nt- and 24 nt-phasiRNAs were systemically characterized using PhaseTank packet [77]. PhasiRNAs with *P*-value < 0.05 and |log2 (fold change ratio)| > 1 were considered as differentially expressed phasiRNAs.

### Characterization of lncRNAs

To investigate the conservation of lncRNAs, all the lncRNAs sequences were blasted against the genome sequences of *Arabidopsis thaliana* (assembly TAIR10), *Zea mays* (assembly B73 RefGen_v3), *Brachypodium distachyon* (assembly Brachypodium_distachyon_v2.0) and *Sorghum bicolor* (assembly Sorbi1) that download from NCBI with a cut-off E-value <1e-10 by using BLASTN. The lncRNAs with more than 20% sequence matched to other genomes were defined as conversed lncRNAs. Additionally, we also conducted the lncRNAs associated with repetitive element by Repeat Masker (http://www.repeatmasker.org/; based on the Repeat Database namely repeatmaskerlibraries-20,160,829 from http://www.girinst.org/) with the ‘slow’ option. To identify the lncRNAs that may act as precursors of miRNAs or phasiRNAs, the small RNAs were aligned to the identified lncRNAs by following the model of Boerner and McGinnis [29]. LncRNAs as targets of miRNAs were predicted by psRNATarget [31], with expectation ≤3 [47]. The target mimics were predicted according to the method of Meng et al. [78].

### Target gene prediction and functional analysis of lncRNAs

LncRNAs may play cis- and trans-acting regulation on the genes [47]. Two algorithms were used for cis- and trans- target prediction. The first algorithm was used by python script to detect potential cis target genes (differentially expressed genes) that are physically close to lncRNAs (differentially expressed lncRNAs). The genes transcribed within a 100 kb window upstream or downstream of lncRNAs were considered as potential cis target genes [79]. The second algorithm was used to identify potential trans targets based on RNA sequence complementarity (mRNA-lncRNA) and RNA duplex energy prediction [80, 81]. Based on the RNA duplex energy (Free energy < − 50) assessed by RIsearch algorithms [82], the differentially expressed genes and lncRNAs were considered as potentially involved in trans-interaction. Then, functional analysis of the lncRNAs cis/trans targets were done by using the AgriGO [83]. Significance was expressed as a *P*-value < 0.05. Protein-protein interaction networks were performed using STRING [84].

### Quantitative real-time PCR (qRT-PCR) analysis

Reverse-transcribed of total RNAs isolated from ovaries and anthers, and qRT-PCR assay were performed according to Li et al. [85]. The qRT-PCR products of each candidate lncRNAs were Sanger sequenced by BGI Genomics, and the amplified fragments were matched to the lncRNAs. The specificity of the amplified fragments was checked using the generated melting curve. GAPDH was used as an internal control gene. All qRT-PCR amplifications were carried out in three replications, and the results are presented as the mean ± standard deviations. The *2*^*–ΔΔCT*^ method was employed to calculate the relative expression level [86]. The primers were designed by Primer 5.0 software, and are listed in Additional file 3: Table S19.

### 5′- and 3′-rapid amplification of cDNA ends (RACE) assay

Full-length amplification of lncRNA was performed with SMARTer RACE 5′/3′ Kit (Clontech, USA) according to the manufacturer’s instructions. PCR was performed with Universal Primer A Mix and gene-specific primers using 5′/3′-cDNA as the template (Additional file 3: Table S19). The 5′- and 3′-RACE products were gel-purified and cloned. At least 12 clones were selected for Sanger sequencing to obtain the longest fragment. Finally, long distance PCR using primers designed from the extreme 5′ and 3′ ends of the candidate lncRNA and the 5′-cDNA as a template to generating full-length lncRNA.

### Selection of the target site and plasmid construction

Specific gRNA targeting lncRNA57811, lncRNA55980, and lncRNA130461 were selected using the online tool (http://skl.scau.edu.cn/targetdesign/). The gRNA expression cassettes were generated by overlapping PCR. The target sequences were ligated between the corresponding promoter and sgRNA by the first PCR. The second PCR was used to induce a *BsaӀ* restriction site. The amplified fragments were then assembled on the pYLCRISPR/Cas9Ubi-H binary plasmid. To construct the overexpressed lncRNA57811 vector, the cDNA of lncRNA57811 were obtained and fused into the POX vector (digestion by *HindIII* and *BamHI*) using Hieff Clone® Plus One Step Cloning Kit (Hieff, Shanghai, China). To construct lncRNA57811-Pro::GUS vector, 2.0 kb upstream of ATG sequence was amplified from Taichung 65 genomic DNA and cloned into pCAMBIA1305.1. These constructs were transferred to *Agrobacterium* EHA105, and then were transformed into rice callus (Taichung 65). The specific primers are listed in Additional file 3: Table S19.

## Supplementary information

**Additional file 1 Fig. S1** Identification of the lncRNAs in the rice reproductive tissues. **Fig. S2** Characteristics of lncRNAs in rice reproductive tissues. **Fig. S3** Sequence alignment results between lncRNAs obtained by RACE assay and transcripts predicted by transcriptome. **Fig. S4** Phylogenic tree analysis of lncRNAs based on the rice dataset. **Fig. S5** Anther and ovary-preferred lncRNAs in diploid (A) and autotetraploid rice (B). **Fig. S6** Venn analysis of DEL-anther and DEL-ovary in autotetraploid rice. **Fig. S7** Classification of differentially expressed lncRNAs in anther (A) and ovary (B) of autotetraploid rice. **Fig. S8** Transposable elements (TEs) associated with lncRNAs in autotetraploid rice. **Fig. S9** Validation of the transposable elements (TEs) associated genes and TEs-lncRNAs in autotetraploid compared to diploid rice. **Fig. S10** Venn analysis of the predicted targets of differentially expressed lncRNAs (DEL) and differentially expressed miRNAs (DEM). **Fig. S11** Protein-protein interactions between the EMC meiosis-related targets of differentially expressed lncRNAs. **Fig. S12** Protein-protein interactions between the PMC meiosis-related targets of differentially expressed lncRNAs. **Fig. S13** Validation of the differentially expressed lncRNAs (DEL) in autotetraploid and diploid rice. **Fig. S14** Validation of the targets of differentially expressed lncRNAs in autotetraploid and diploid rice. **Fig. S15** Agronomic traits of wild type and mutants in T1 generation. **Fig. S16** Phenotypes of *LOC_Os12g41350*-CR and *LOC_Os12g41350*-OE in T1 generation.

**Additional file 2 Table S1.** Characteristics of all lncRNAs identified in this study. **Table S2.** Conservation of lncRNAs in other plant species. **Table S3.** Differentially expressed lncRNAs (DEL) in anther and ovary of Taichung 65-4x compared to Taichung 65. **Table S4.** The lncRNAs selected by aligment assay compared to a previous study. **Table S5.** Summary of known and predicted miRNAs detected in this study. **Table S6.** Overview of 21 nt- and 24 nt-phasiRNAs detected in this study. **Table S7.** LncRNAs corresponding to miRNA precursors. **Table S8.** LncRNAs corresponding to phasiRNA precursors. **Table S9.** A list of lncRNAs predicted to be the targets of miRNAs. **Table S10.** Transposable elements-associated with differentially expressed lncRNAs in autotetraploid rice.

**Additional file 3 Table S11.** Classification of differentially expressed lncRNAs related to transposable elements (TEs) in autotetraploid rice. **Table S12.** The potential target genes of differentially expressed lncRNAs detected by cis-regulation. **Table S13.** The potential target genes of differentially expressed lncRNAs detected by trans-regulation. **Table S14.** GO (Gene Ontology) analysis of predicted targets of differentially expressed lncRNAs. **Table S15.** Overall information about the predicted targets of differentially expressed miRNAs (DEM). **Table S16.** GO (Gene Ontology) analysis of predicted targets of differentially expressed lncRNAs and differentially expressed miRNAs. **Table S17.** The most important lncRNA-target pairs related to PMC meiosis in autotetraploid rice. **Table S18.** The important lncRNA-target pairs related to EMC meiosis in autotetraploid rice. **Table S19.** A list of primers used in the present study. **Table S20.** The information about ten lncRNAs detected by RACE in the present study.

## Data Availability

Transcriptome and small RNA sequencing data are available from the NCBI under the accession numbers PRJNA395615 (https://www.ncbi.nlm.nih.gov/bioproject/PRJNA395615) and PRJNA396435 (https://www.ncbi.nlm.nih.gov/bioproject/PRJNA396435). The accession numbers of ten lncRNAs are MN481253 (lncRNA57811), MN481254 (lncRNA130461), MN481255 (lncRNA55980), MN481256 (lncRNA91337), MN481257 (lncRNA68868), MN481258 (lncRNA111916), MN481259 (lncRNA130471), MN481260 (lncRNA45430), MN481261 (lncRNA13598) and MN481262 (lncRNA121908). The information about lncRNAs are also available in Additional file 3: Table S20. All data supporting the conclusions described here are provided in tables, figures and additional files.

## Abbreviations

DEG: differentially expressed genes
DEL: differentially expressed lncRNAs
DEM: differentially expressed miRNAs
EMC: embryo sac mother cell
PMC: pollen mother cell
