# Supplementary material for: Global identification and analysis revealed differentially expressed lncRNAs associated with meiosis and low fertility in autotetraploid rice
Source: BMC Plant Biol. 2020 Feb 19;20:82. doi: 10.1186/s12870-020-2290-0 (PMC7032005; doi:10.1186/s12870-020-2290-0)
Supplement: Supplementary file 1 — Additional file 1 Fig. S1 Identification of the lncRNAs in the rice reproductive tissues. Fig. S2 Characteristics of lncRNAs in rice reproductive tissues. Fig. S3 Sequence alignment results between lncRNAs obtained by RACE assay and transcripts predicted by transcriptome. Fig. S4 Phylogenic tree analysis of lncRNAs based on the rice dataset. Fig. S5 Anther and ovary-preferred lncRNAs in diploid (A) and autotetraploid rice (B). Fig. S6 Venn analysis of DEL-anther and DEL-ovary in autotetraploid rice. Fig. S7 Classification of differentially expressed lncRNAs in anther (A) and ovary (B) of autotetraploid rice. Fig. S8 Transposable elements (TEs) associated with lncRNAs in autotetraploid rice. Fig. S9 Validation of the transposable elements (TEs) associated genes and TEs-lncRNAs in autotetraploid compared to diploid rice. Fig. S10 Venn analysis of the predicted targets of differentially expressed lncRNAs (DEL) and differentially expressed miRNAs (DEM). Fig. S11 Protein-protein interactions between the EMC meiosis-related targets of differentially expressed lncRNAs. Fig. S12 Protein-protein interactions between the PMC meiosis-related targets of differentially expressed lncRNAs. Fig. S13 Validation of the differentially expressed lncRNAs (DEL) in autotetraploid and diploid rice. Fig. S14 Validation of the targets of differentially expressed lncRNAs in autotetraploid and diploid rice. Fig. S15 Agronomic traits of wild type and mutants in T1 generation. Fig. S16 Phenotypes of LOC_Os12g41350-CR and LOC_Os12g41350-OE in T1 generation. [file 12870_2020_2290_MOESM1_ESM.pdf]

Additional file 1

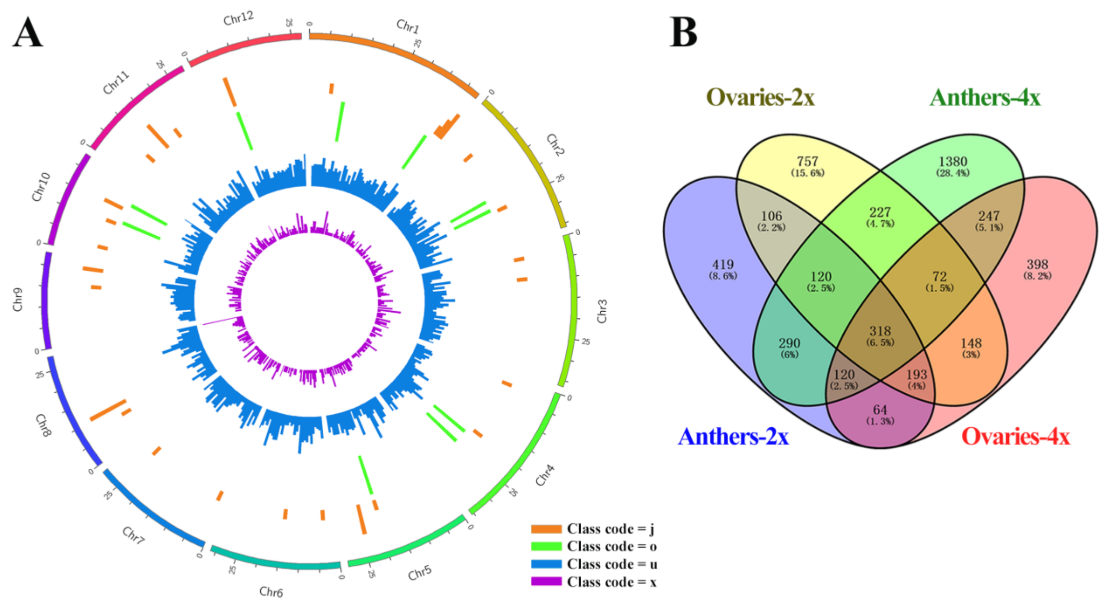

Fig. S1 Identification of the lncRNAs in the rice reproductive tissues. (A) Distribution of lncRNAs along each chromosome by circus. Class codes ‘u’ and ‘x’ indicate the lncRNAs in the intergenic and antisense sequence, respectively. ‘o’ and ‘j’ indicate the lncRNAs overlapped with known exons. (B) Venn analysis of each tissue in diploid and autotetraploid rice. ‘4x’ and ‘2x’ indicate autotetraploid and diploid rice, respectively.

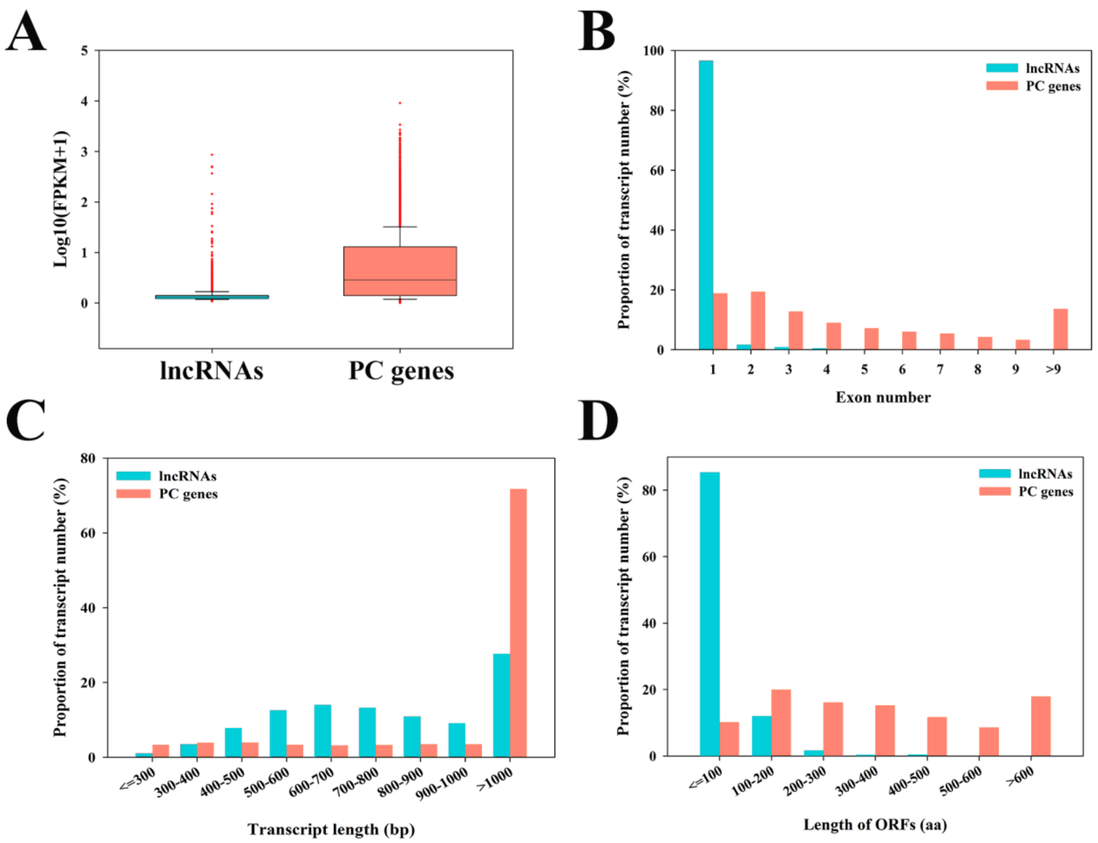

Fig. S2 Characteristics of lncRNAs in rice reproductive tissues. (A) The expression levels of lncRNAs and protein-coding genes (PC genes). (B) The number of exons in the lncRNAs and protein-coding genes. (C) Transcript length distributions in the lncRNAs and protein-coding genes. (D) Length of open reading frames (ORFs) in the lncRNAs and protein-coding genes.

lncRNA57811

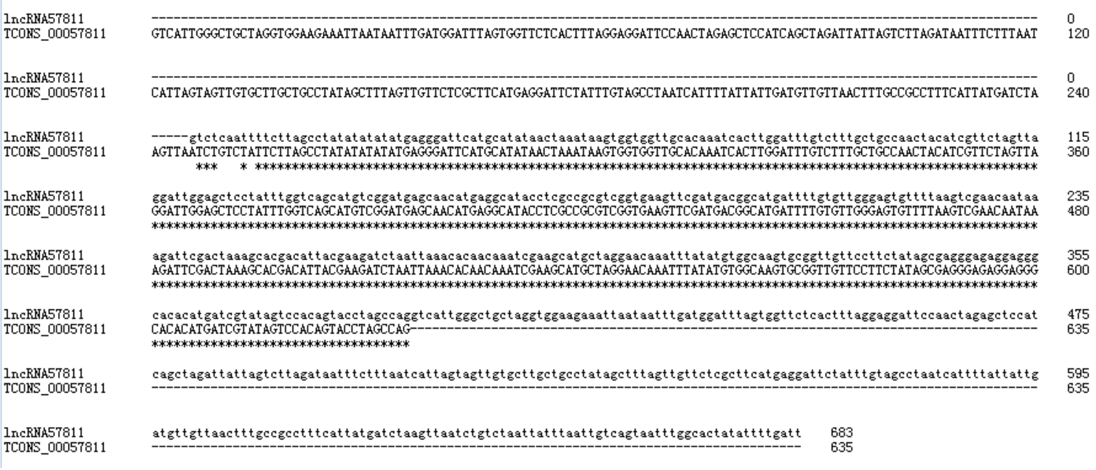

lncRNA130461

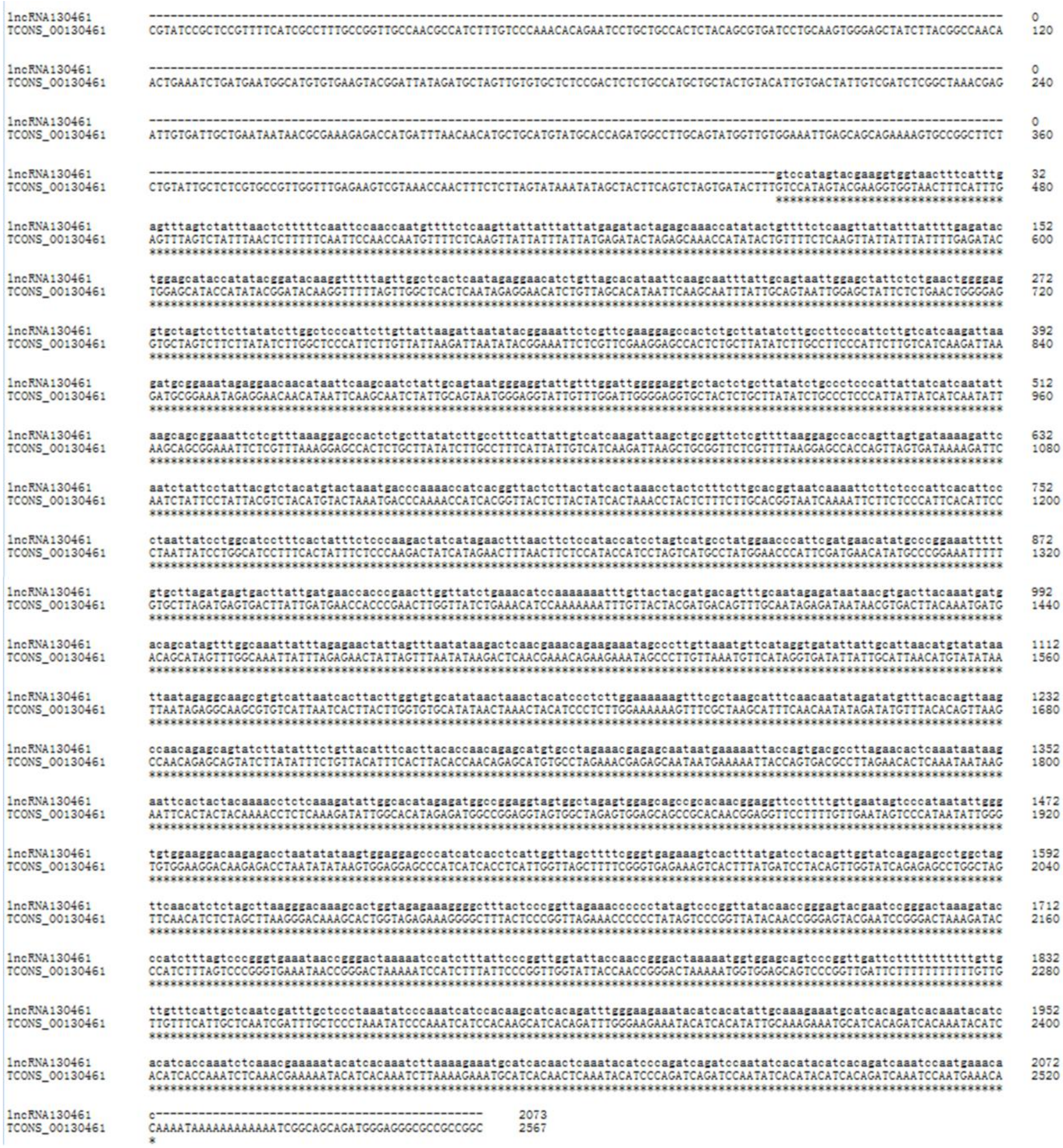

lncRNA68868

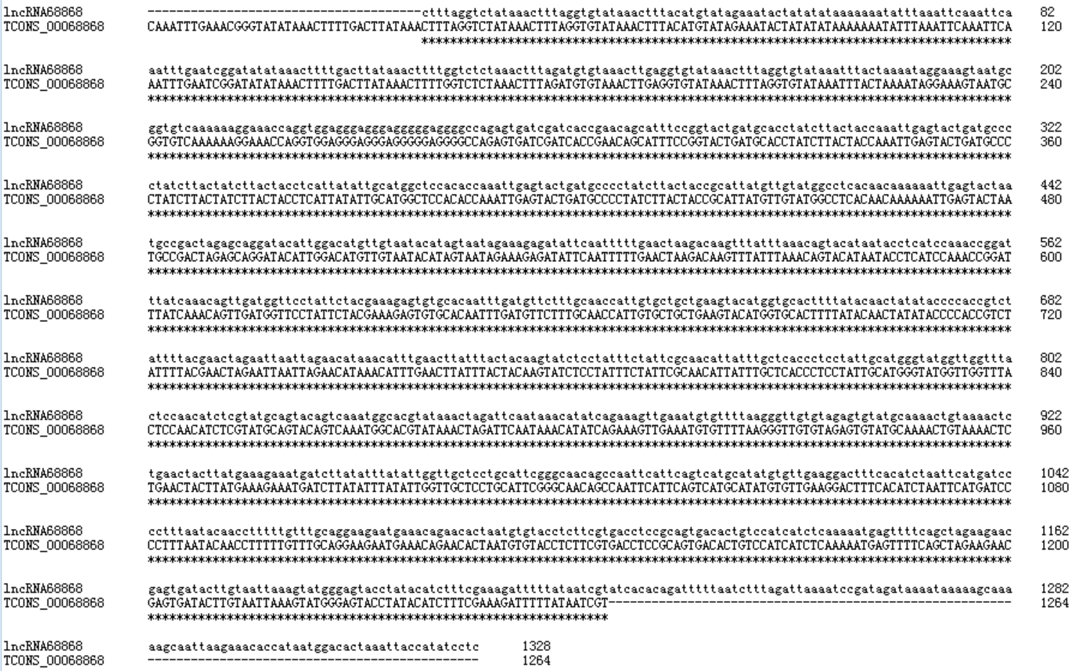

lncRNA91337

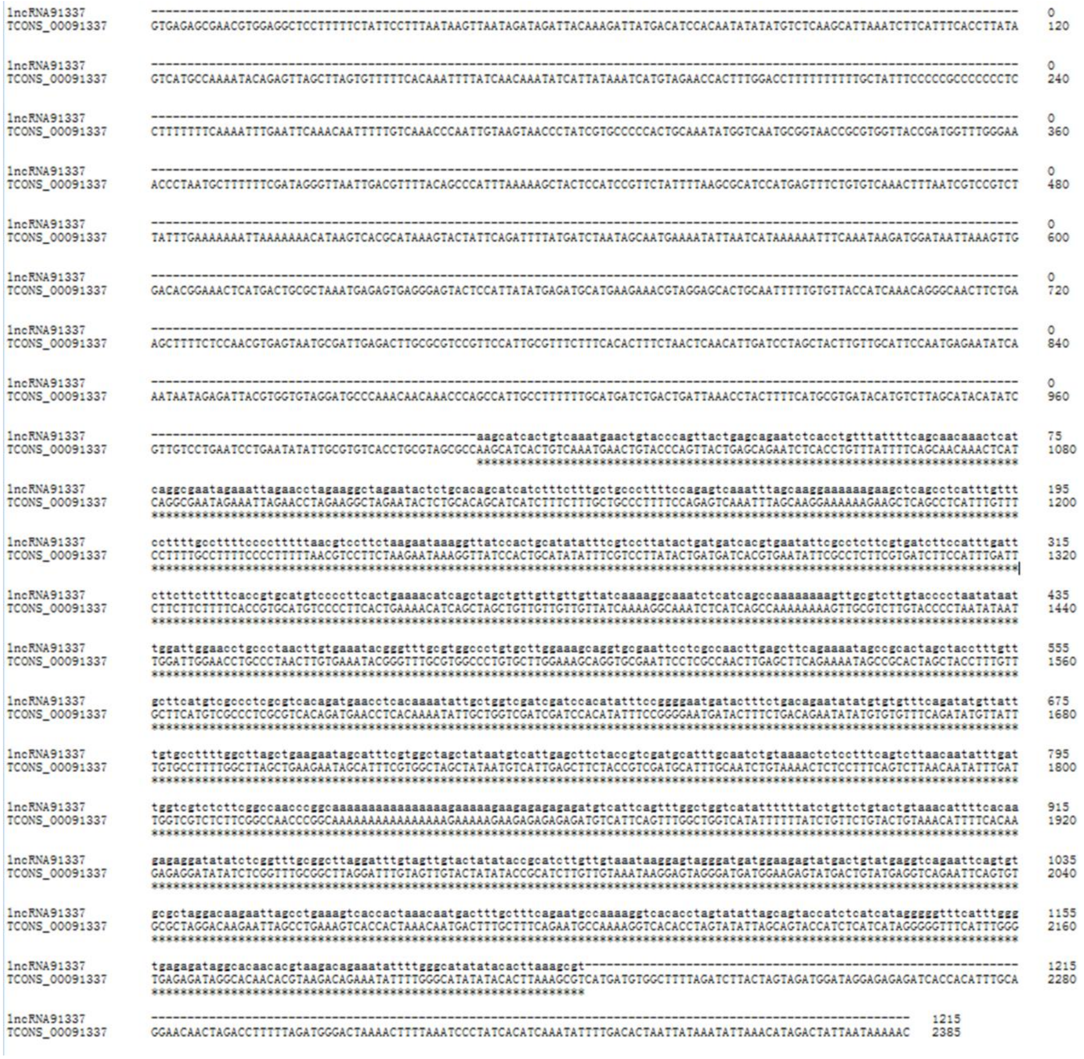

lncRNA130471

```
lncRNA130471      ------------------------------------------------------------------------------------------------------------------------    0
TCONS_00130471    CTTGAATTTCAAGATTGTTCCCGTCCGATCTCTAACTAAACCATGCGCGAAACGAGTGAGCCGAGGAGCAGAGGATCTGCATGTATGTAGCACTGCTGATCGTCGAATTCACGCATGATA    120

lncRNA130471      ------------------------------------------------------------------------------------------------------------------------    0
TCONS_00130471    GACGACGGGTAGCACCTCAGCATGCAAAAGAAGTTCAGAATAATGAAGCATAACACAACTTGTATGGTTTCAAAATAACCACGTAGTGCCTCTTCTATTCTTCATGTAATCCTCGCAATT    240

lncRNA130471      ------------------------------------------------------------------------------------------------------------------------    0
TCONS_00130471    AACTGTGGGATCGATTAACAGATAAAAGGTCTTTTTTAAAAAAAAAAAATTCAATAGTTAAAGCCCTGCCGGCTTTGCTCTGAATTAGTCTCCTGCTGTCGCGGCTAAATGTTATGCTCAA    360

lncRNA130471      ------------------------------------------------------------------------------------------------------------------------    0
TCONS_00130471    CGAGCCGAGGCCTTTCAGACCCATCCCCCACTATGAGATAACTAAGTTGAGAAAGTCATGAATCAAATGTGAGTCATCTCCGTTTATTGTCATATGAATGTAATGATTGATTTAACAATA    480

lncRNA130471      ---------------------------------atagtgtctccatcttatcagtctccgttcggtgagatattggggcaaaccatgcaatggtcacaaggtgaatccacgagagctttg    87
TCONS_00130471    TAAATGGGAGAGAAACTTCTAAATGATAATTGTATAGTGTCTCCATCTTATCAGTCTCCGTTCGGTGAGATATTGGGGCAAACCATGCAATGGTCACAAGGTGAATCCACGAGAGCTTTG    600
                                                   ***************************************************************************************

lncRNA130471      tttcccgatatttcagtaagaggttgggataactcgattagatgaagattatgttgatggcccaactacaactgtccaaggacgctacgccttaccaaccaatacactatatccaacagt    207
TCONS_00130471    TTTCCCGATATTTCAGTAAGAGGTTGGGATAACTCGATTAGATGAAGATTATGTTGATGGCCCAACTACAACTGTCCAAGGACGCTACGCCTTACCAACCAATACACTATATCCAACAGT    720
                  ************************************************************************************************************************

lncRNA130471      tgttgtgaacttatggtgtacgacaactcggccatgaggacactcgtcttgcaagcaatcgaaaaacaagtagaacaaacactgaattgttagatgaatatggaagtttcactagaaaaa    327
TCONS_00130471    TGTTGTGAACTTATGGTGTACGACAACTCGGCCATGAGGACACTCGTCTTGCAAGCAATCGAAAAACAAGTAGAACAAACACTGAATTGTTAGATGAATATGGAAGTTTCACTAGAAAAA    840
                  ************************************************************************************************************************

lncRNA130471      taacaatattgtagggttcaaacagatgggctgtttagccaacacgcacacggtaaacaagtagcaatttgctaactctaagcatcaaaaccaattgttattgacggtggcaaggtatac    447
TCONS_00130471    TAACAATATTGTAGGGTTCAAACAGATGGGCTGTTTAGCCAACACGCACACGGTAAACAAGTAGCAATTTGCTAACTCTAAGCATCAAAACCAATTGTTATTGACGGTGGCAAGGTATAC    960
                  ************************************************************************************************************************

lncRNA130471      gaaaatgaggagaacaaccatacggggtgttggatttgtcctctaacctgcaatgtgtccctaatggaatcaactttacagcccctgcgatgcgtccgtaatagaaccaacttgatgcac    567
TCONS_00130471    GAAAATGAGGAGAACAACCATACGGGGTGTTGGATTTGTCCTCTAACCTGCAATGTGTCCCTAATGGAATCAACTTTACAGCCCCTGCGATGCGTCCGTAATAGAACCAACTTGATGCAC    1080
                  ************************************************************************************************************************

lncRNA130471      aacccattaggcccaaataagatgatgcattacatttcacaaaaaagacgtcgatagtaaaatataatgtttgtggccgctccagagcagatggacatgaggccgaatccatttgaaaat    687
TCONS_00130471    AACCCATTAGGCCCAAATAAGATGATGCATTACATTTCACAAAAAAGACGTCGATAGTAAAATATAATGTTTGTGGCCGCTCCAGAGCAGATGGACATGAGGCCGAATCCATTTGAAAAT    1200
                  ************************************************************************************************************************

lncRNA130471      ggcttgataagcttttcatgcaatacttgcacccaaatcaaagtttgcatgaagtcgtggcagtcatcacaagttggcactgtcctaccattcgaatatagaccgcgcgaattctctccc    807
TCONS_00130471    GGCTTGATAAGCTTTTCATGCAATACTTGCACCCAAATCAAAGTTTGCATGAAGTCGTGGCAGTCATCACAAGTTGGCACTGTCCTACCATTCGAATATAGACCGCGCGAATTCTCTCCC    1320
                  ************************************************************************************************************************

lncRNA130471      cttttaatcctctcaatcattctaagtaaaacaagagtgcacgcgtcttcggtataaaaaatatgaacatgaactcaagaataaactcacctgatagttgaattgacgtgcacgagcacg    927
TCONS_00130471    CTTTTAATCCTCTCAATCATTCTAAGTAAAACAAGAGTGCACGCGTCTTCGGTATAAAAAATATGAACATGAACTCAAGAATAAACTCACCTGATAGTTGAATTGACGTGCACGAGCACG    1440
                  ************************************************************************************************************************

lncRNA130471      tgtaataggaccatgagattgtacttgtggagacttatgttagacgtgtacatggaggagacggataacaagtcaaagtggtggatgttatagcttttggaattaagtggcctttgtggc    1047
TCONS_00130471    TGTAATAGGACCATGAGATTGTACTTGTGGAGACTTATGTTAGACGTGTACATGGAGGAGACGGATAACAAGTCAAAGTGGTGGATGTTATAGCTTTTGGAATTAAGTGGCCTTTGTGGC    1560
                  ************************************************************************************************************************

lncRNA130471      cgtagtggagaacctactttagttttcagatataaactgatgtttttcattaatttttattttcatgtgtaaattgagtttttttaaaaaaatgatttataaacagcgcacgaggcatg    1167
TCONS_00130471    CGTAGTGGAGAACCTACTTTAGTTTTCAGATATAAACTGATGTTTTTCATTAATTTTTATTTTCATGTGTAAATTGAGTTTTTTTAAAAAAATGATTTATAAACAGCGCACGAGGCATG    1680
                  ************************************************************************************************************************

lncRNA130471      gattttcatgcttacataatcagcgaataaaaaacctctatatttgaggaaaacacctccc--------------------------------------------------------    1228
TCONS_00130471    GATTTTCATGCTTACATAATCAGCGAATAAAAAACCTCTATATTTGAGGAAAACACCTCCCTATTTATGACACCTCCCTATTTATTATCATATTATCTAATTCTACTATACATGATC    1797
                  *************************************************************
```

lncRNA13598

```
lncRNA13598       ctcgatcggatttgttcatcaaaatagaacctcacttatacatgcacaccatttcgatttctcctatcaatatttaattcaattttgtactatggttcatatttaggatatagtagcat    120
TCONS_00013598    ------------------------------------------------------------------------------------------------------------------------    0

lncRNA13598       tatttacccagtttgactctctaacacatttttttgttgctgtagtttcccatcctcttttgtttcccaggttttaggcccacggttactgacttagttgatttttactcttgatttgtc    240
TCONS_00013598    ------------------------------------------------------------------------------------------------------------------------    0

lncRNA13598       tcttaagcggttgaagggtatggaaaacaatatcatatacatccattggttgatttgtcacccaatcagatctaagaatgtatgggcatgttaagccgatgaagttttttttaattggtat    360
TCONS_00013598    ------------------------------------------------------------------------------------------------------------------------    0

lncRNA13598       tcaattatgatctttagctggtgaagttatatcacggtatatataaggtgctggtgcaactgatctggacatatttgtgactgaatctgcttagtaatattggtatttcggaagttttct    480
TCONS_00013598    ------------------------------------------------------------------------------------------------------------------------    0

lncRNA13598       gaaagctatgaacactgcaaggagttaacgaaggatgagaacttaattatatattttagaggtaatccttttcccaactccgatagctaagcaggaatttgttctaccattctctatgat    600
TCONS_00013598    -------------------------------------------------------------------------------------------------------------------ATGAT    5
                                                                                                                                     *****

lncRNA13598       cgtttaacagtttagttatgattcccagctaacagtgataattggactatgtatgtagatgtttgaatataaaatgagagtctttttagtgatggttatcaattttgtaattgtatggta    720
TCONS_00013598    CGTTTAACAGTTTAGTTATGATTCCCAGCTAACAGTGATAATTGGACTATGTATGTAGATGTTTGAATATAAAATGAGAGTCTTTTTAGTGATGGTTATCAATTTTGTAATTGTATGGTA    125
                  ************************************************************************************************************************

lncRNA13598       gtcatggaccagatttgctacatgccttgactgcacattcctggcgtatcatatgacaacatataaataatacggagtaattgttacttccggctaaatgtacgtttcttaccgcaataa    840
TCONS_00013598    GTCATGGACCAGATTTGCTACATGCCTTGACTGCACATTCCTGGCGTATCATATGACAACATATAAATAATACGGAGTAATTGTTACTTCCGGCTAAATGTACGTTTCTTACCGCAATAA    245
                  ************************************************************************************************************************

lncRNA13598       aaatatgatatttcagcaacttattcatgaactgattactgattagcattctattgcaagattaactactgaggtactcctctaaaaaaactactgaggtactaaaaccgagctaaaaag    960
TCONS_00013598    AAATATGATATTTCAGCAACTTATTCATGAACTGATTACTGATTAGCATTCTATTGCAAGATTAACTACTGAGGTACTCCTCTAAAAAAACTACTGAGGTACTAAAACCGAGCTAAAAAG    365
                  ************************************************************************************************************************

lncRNA13598       gatcagtgagggagagctgtactacaaaatacattatgcagaatacaaggctaccaagtcaatgaggatatcagttccagtacaccacctatatattttccttccagtctcagttcatat    1080
TCONS_00013598    GATCAGTGAGGGAGAGCTGTACTACAAAATACATTATGCAGAATACAAGGCTACCAAGTCAATGAGGATATCAGTTCCAGTACACCACCTATATATTTTCCTTCCAGTCTCAGTTCATAT    485
                  ************************************************************************************************************************

lncRNA13598       agatatttttttagaaaggttcatatggacattaaacatataattctgcataattttattgatggtgtacatatttttcattcttcagcaacgaggtaacagactttaattttattgttcc    1200
TCONS_00013598    AGATATTTTTTAGAAAGGTTCATATGGACATTAAACATATAATTCTGCATAATTTTATTGATGGTGTACATATTTTTCATTCTTCAGCAACGAGGTAACAGACTTTAATTTTATTGTTCC    605
                  ************************************************************************************************************************

lncRNA13598       aatactaggtctatccggtcagcacctatgtatctcacctgcaattctgttgcatttttaaccacggctacctaagtttagatagctcatcacataatttttactccatccattccataa    1320
TCONS_00013598    AATACTAGGTCTATCCGGTCAGCACCTATGTATCTCACCTGCAATTCTGTTGCATTTTTAACCACGGCTACCTAAGTTTAGATAGCTCATCACATAATTTTTACTCCATCCATTCCATAA    725
                  ************************************************************************************************************************

lncRNA13598       cataaggaatctattgtcattataaaggttttacaaagtcaatcacttaggattattttttttctttcctcatatgctactatgctcatggtgcatctttgaaatttatttattattccc    1440
TCONS_00013598    CATAAGGAATCTATTGTCATTATAAAGGTTTTACAAAGTCAATCACTTAGGATTATTTTTTTTCTTTCCTCATATGCTACTATGCTCATGGTGCATCTTTGAAATTTATTTATTATTCCC    845
                  ************************************************************************************************************************

lncRNA13598       cgcagcaacgcatagggtattatctagtttttgaatgcttccatcccaaacgttctttgctgagacggtctgtaacccaattcgcactgatattttggtataaccgatcagaagttgaaa    1560
TCONS_00013598    CGCAGCAACGCATAGGGTATTATCTAGTTTTTGAATGCTTCCATCCCAAACGTTCTTTGCTGAGACGGTCTGTAACCCAATTCGCACTGATATTTTGGTATAACCGATCAGAAGTTGAAA    965
                  ************************************************************************************************************************

lncRNA13598       gttgaagcatagtccattcactgcaagatgttaaataaccaacagatagaaatttaattatcatatctataatagatgatatacacagtcaagttaaataacctttttttatctgattatt    1680
TCONS_00013598    GTTGAAGCATAGTCCATTCACTGCAAGATGTTAAATAACCAACAGATAGAAATTTAATTATCATATCTATAATAGATGATATACACAGTCAAGTTAAATAACCTTTTTTTATCTGATTATT    1085
                  ************************************************************************************************************************

lncRNA13598       atattatatactctctttgtcccaaattataaggtgtatatttttacacggtttttaatgacatattttgataatcaatttattctatattatgttctcaacaaatatgaaattagcatc    1800
TCONS_00013598    ATATTATATACTCTCTTTGTCC--------------------------------------------------------------------------------------------------    1107
                  **********************

lncRNA13598       acataaaaatatatttagcatatatatgattagtatgattattaattaaaagttagcgagtttgatttttttcttaaaaaaggacaccttataatttgggacgaatggagtataaggccca    1920
TCONS_00013598    ------------------------------------------------------------------------------------------------------------------------    1107

lncRNA13598       aattaagctattaaggcactctttgtttagatttgtaagttagcttataagg        1972
TCONS_00013598    ----------------------------------------------------        1107
```

lncRNA11916

```
lncRNA111916      ------------------------------------------------------------------------------------------------------------------------   0
TCONS_00111916    GTTCGATGCACCAAACATGTATGGCCAAATCCGTATGTCAACTAAGTTGTATAAGACTCATCAAACTTGATTTTTTTCCTTTTTTTTTTCCGGCAATATTTTCCCCCTTTGGTTGAGAAG   120

lncRNA111916      ------------------------------------------------------------------------------------------------------------------------   0
TCONS_00111916    TCATGCATGAAGCTAATGTGGGAACTACATCACTAATGCATGATCCCCCAACAAAAGCAGAATGAATTTATTGGCATGGATATTGTGGATTATTTTTCCAGTATAAATGAGAGAGCAACC   240

lncRNA111916      ------------------------------------------------------------------------------------------------------------------------   0
TCONS_00111916    TTGTACAAGCAATATATATTTAGCCAATCAAACATTTTGGCATTCAACTAAGTGGTTTTTCACAAGTCCTATTTGCTAGCAGATATATTTGCAGGGAACTAGTATCTGAAGGTGTTGATA   360

lncRNA111916      ------------------------------------------------------------------------------------------------------------------------   0
TCONS_00111916    GGGTTTAATTTATCTGATGGTTCTGACCATATTAAATAGAGGGTCTATTTTATCGTTTGTCTGTCTTGGAGCCGAACAAGTAAGGCGTTCGGCAATTTTTCACTTCAAAGTTCTTTACGC   480

lncRNA111916      ------------------------------------------------------------------------------------------------------------------------   0
TCONS_00111916    CCTGAGAAAGAGCATACCTATGTGCGATTGAGTCGTAGGTTGCGACCGAGTTTATCAAACCATTATCACTTTTGCCTTTTTCTTTTTTTAGAATTAGGGATGAAAGAAAAAACACAAAGA   600

lncRNA111916      ------------------------------------------------------------------------------------------------------------------------   0
TCONS_00111916    AAGAGATCGGGAAACTTTATTTATAACCGGAGAAATGGGATATTAGAGTAAACCACATGAAAGTTAGGTCTATGTGAAACAGAACGGGAAACTTTATTTATGACCAGATAAATAGGATAT   720

lncRNA111916      ------------------------------------------------------------------------------------------------------------------------   0
TCONS_00111916    TACGGTAAACTACACAAAAATTAGACCTATGTACCACACAGAAGTTAGGCCTATGTACGATTAGTTCACCTAAACAAATGTAAATGTTTATGTCCATATTGATCTGGATTGCTTTTGTGT   840

lncRNA111916      ------------------------------------------------------------------------------------------------------------------------   0
TCONS_00111916    CACATAGTTGTACAGTTCTTGATTGGAAAAGAAGAATTCTCCAACACATGTGTGGGAGATTTTGATGTTACTAATATAATTGGTTCAATACAATCTTGGCATATATTTCTCATTGTGCTA   960

lncRNA111916      ----------------------------------------------------------------------actattttctcaaataggcatgattattttgcaatataaatgtgatcaaa   50
TCONS_00111916    CTAACATAATTGGTAAAATACAGCCATGGTAGCACCTGTTTGGCCCAATTGCATGTGCATCTTGTGGAGTACTATTTTCTCAAATAGGCATGATTATTTTGCAATATAAATGTGATCAAA   1080
                                                                                        **************************************************

lncRNA111916      ttgacgtcgtctaggtgtataatcatcgtctaggattcaggtgcatggtaagaccatctcaagtactgataaaaactatcctatatatttatatagttgatcccacctactgtagttatt   170
TCONS_00111916    TTGACGTCGTCTAGGTGTATAATCATCGTCTAGGATTCAGGTGCATGGTAAGACCATCTCAAGTACTGATAAAAACTATCCTATATATTTATATAGTTGATCCCACCTACTGTAGTTATT   1200
                  ************************************************************************************************************************

lncRNA111916      attgctaatttcatgtcctctcgtgcagccacatcatcacttttatcttcatcgttagatgaatctatatctttgatgtcagcagttggatagaattctctcccatgatacagtccggca   290
TCONS_00111916    ATTGCTAATTTCATGTCCTCTCGTGCAGCCACATCATCACTTTTATCTTCATCGTTAGATGAATCTATATCTTTGATGTCAGCAGTTGGATAGAATTCTCTCCCATGATACAGTCCGGCA   1320
                  ************************************************************************************************************************

lncRNA111916      ataaacaaccaattcatgagcagtcgagtgcgagccaccacagccgcaatacgcaagcgagcgcgaataccagtctccacaattccattaggcatggttgtggtgatagagcttctgcac   410
TCONS_00111916    ATAAACAACCAATTCATGAGCAGTCGAGTGCGAGCCACCACAGCCGCAATACGCAAGCGAGCGCGAATACCAGTCTCCACAATTCCATTAGGCATGGTTGTGGTGATAGAGCTTCTGCAC   1440
                  ************************************************************************************************************************

lncRNA111916      tggatgagaattgtaattggcagcgaagcccggccgtcaacctgtcctcctgcctcccctatcgaaactaccagacgaccactcgactttgataactacaagaggcatcgatcttttgtt   530
TCONS_00111916    TGGATGAGAATTGTAATTGGCAGCGAAGCCCGGCCGTCAACCTGTCCTCCTGCCTCCCCTATCGAAACTACCAGACGACCACTCGACTTTGATAACTACAAGAGGCATCGATCTTTTGTT   1560
                  ************************************************************************************************************************

lncRNA111916      ttaagagaagggtatttttttttacctggcctctatatacaatcggatatatacggctttttaaattagaaacttagcctattaaataaggaacttagctctcaaataaccaaatttgaaa   650
TCONS_00111916    TTAAGAGAAGGGTATTTTTTTTTACCTGGCCTCTATATACAATCGGATATATACGGCTTTTTAAATTAGAAACTTAGCCTATTAAATAAGGAACTTAGCTCTCAAATAACCAAATTTGAAA   1680
                  ************************************************************************************************************************

lncRNA111916      ttcgctactatggagatttgaactcaagaccttaggtgctactcatgtcactgcattgataggaacttagcctattaaacaaggcatcaatctgatccaacgagcattgctatta-----   765
TCONS_00111916    TTCGCTACTATGGAGATTTGAACTCAAGACCTTAGGTGCTACTCATGTCACTGCATTGATAGGAACTTAGCCTATTAAACAAGGCATCAATCTGATCCAACGAGCATTGCTATTACCTCT   1800
                  *******************************************************************************************************************

lncRNA111916      ------------------------------------------------------------------------------------------------------------------------   765
TCONS_00111916    TAATTTTACTTCCTTTCGATTAATCTTCCTCTCACCACCATACTATATTGCTGGAGCCTCCCAAGCACATCAACGACAAGCGTGCTCCTCGCTCATAGGACGGCTTCGCTTTTCCTCTCG   1920

lncRNA111916      ------------------------------------------------------------------------------------------------------------------------   765
TCONS_00111916    CTCTGCATAGCACAGGATGCAGAGCAGATCCCCACAGCAACCCTGGTATACATGTTTCTCTTTGTTCTTTGATCAAAATAAAATTGTCTTTTCCTTTTTATTTACTCTTTGCAGCAAACA   2040

lncRNA111916      ------------------------------------------------------------------------------------------------------------------------   765
TCONS_00111916    ATGTTAACAACACCCGCTCATTTCTTTTCAGTTTACACTGTATGTTTGTTCGAGTATATATATGCACCGACTGGGTGTAACCAAACAAGCTCACTCTTTTCAGTATATGTATGTTTGTTC   2160

lncRNA111916      ------------------------------------------------------------------------------------------------------------------------   765
TCONS_00111916    AGGTATATGTATGCTCCTCGCCCGCCAACTTTTTTAGTTTGAGTTAGACCTCAAAATTTCATAGAAATTCCTTCGGATTAAGTTTCAGTATTGTTTAGCTCAATTTCAAATTAATTATTG   2280

lncRNA111916      ------------------------------------------------------------------------------        765
TCONS_00111916    TTCTCGTTGACAAAACTTGCCGATTCAATCAATTGTCTGTGAATCACTCTTCCTCGGTTGGGACAGTACCTATATTAA        2358
```

lncRNA45430

```
lncRNA45430       gatccaattggagagatgaggcattaccatcgaaattgattgattgttaatgttgaactctgactgatattatagtaataaaggtctttttctgatttttttttagggatcaaggtatatt   120
TCONS_00045430    ------------------------------CGAAATTGATTGATTGTTAATGTTGAACTCTGACTGATATTATAGTAATAAAGGTCTTTTTCTGATTTTTTTTTAGGGATCAAGGTATATT   90
                                                  ******************************************************************************************

lncRNA45430       aggttgatgaaacacgatctgaccatcatagtctaaacctgatattatttatcttgagaagttgaactattattagagcgagtgacgagtgagatgttagcaggagctgaaattatgaag   240
TCONS_00045430    AGGTTGATGAAACACGATCTGACCATCATAGTCTAAACCTGATATTATTTATCTTGAGAAGTTGAACTATTATTAGAGCGAGTGACGAGTGAGATGTTAGCAGGAGCTGAAATTATGAAG   210
                  ************************************************************************************************************************

lncRNA45430       gcactattggattcaaagcaacaactttatttccttatgagcattacttcaaaatactatcccatggttgcatcataaaacaaaatattctcaatagtatctagagtcactcaaattgga   360
TCONS_00045430    GCACTATTGGATTCAAAGCAACAACTTTATTTCCTTATGAGCATTACTTCAAAATACTATCCCATGGTTGCATCATAAAACAAAATATTCTCAATAGTATCTAGAGTCACTCAAATTGGA   330
                  ************************************************************************************************************************

lncRNA45430       taaagtgaggtaatataacaccaagaagttcggaaaaattacgaagatttattatgtataatcttaataaaaactctatacgaattattatgtataatcttaatggaaattgtatacatt   480
TCONS_00045430    TAAAGTGAGGTAATATAACACCAAGAAGTTCGGAAAAATTACGAAGATTTATTATGTATAATCTTAATAAAAACTCTATACGAATTATTATGTATAATCTTAATGGAAATTGTATACATT   450
                  ************************************************************************************************************************

lncRNA45430       tggcattttgttcatggtattttacatatttctccctctagacgttttagattggaggcattattgggtagataattaaatatattgaaggatttgcatcttatccatttttgttgatgc   600
TCONS_00045430    TGGCATTTTGTTCATGGTATTTTACATATTTCTCCCTCTAGACGTTTTAGATTGGAGGCATTATTGGGTAGATAATTAAATATATTGAAGGATTTGCATCTTATCCATTTTTGTTGATGC   570
                  ************************************************************************************************************************

lncRNA45430       ttaatcactttttttgttctattatatggaaataagcacctacactacatgctccctccgtggacatatgtttgtcttaaattcgcagtaataggatatgtcacatccggtactgatttgt   720
TCONS_00045430    TTAATCACTTTTTTTGTTCTATTATATGGAAATAAGCACCTACACTACATGCTCCCTCCGTGGACATATGTTTGTCTTAAATTCGCAGTAATAGGATATGTCACATCCGGTACTGATTTGT   690
                  ************************************************************************************************************************

lncRNA45430       tttatattggacagaggagtactacacacccataagttgcaccattaatacacgcttaattagtta---          786
TCONS_00045430    TTTATATTGGACAGAGGAGTACTACACACCCATAAGTTGCACCATTAATACACGCTTAATTAGTTAGAA          759
                  ******************************************************************
```

lncRNA55980

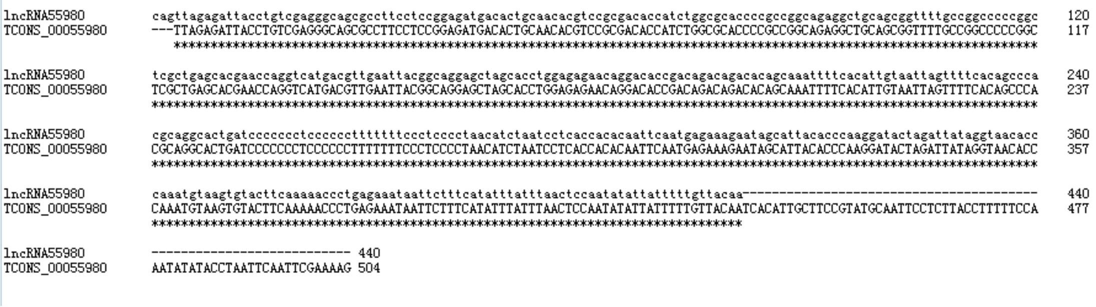

lncRNA121908

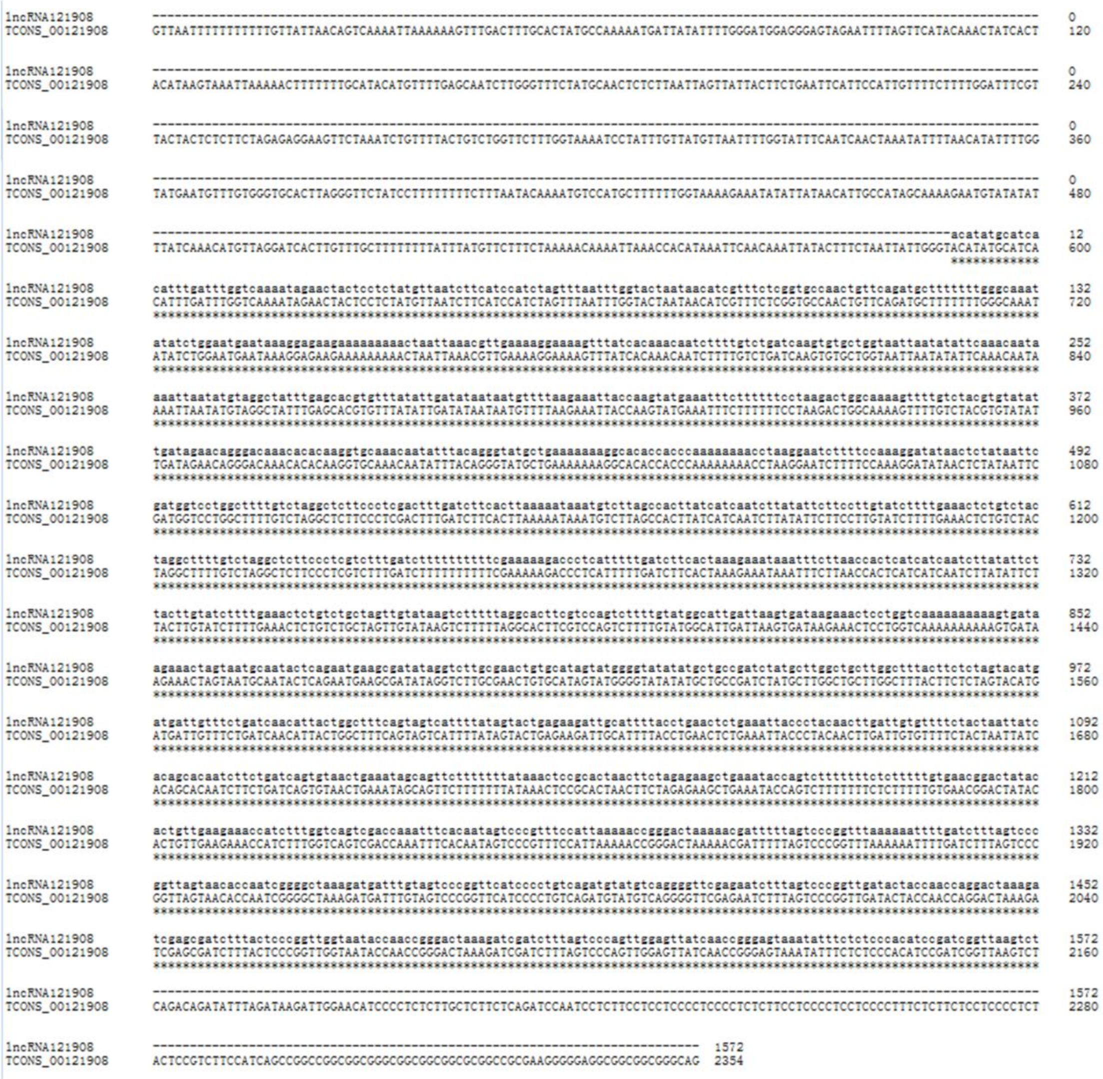

Fig. S3 Sequence alignment results between lncRNAs obtained by RACE assay and transcripts predicted by transcriptome.

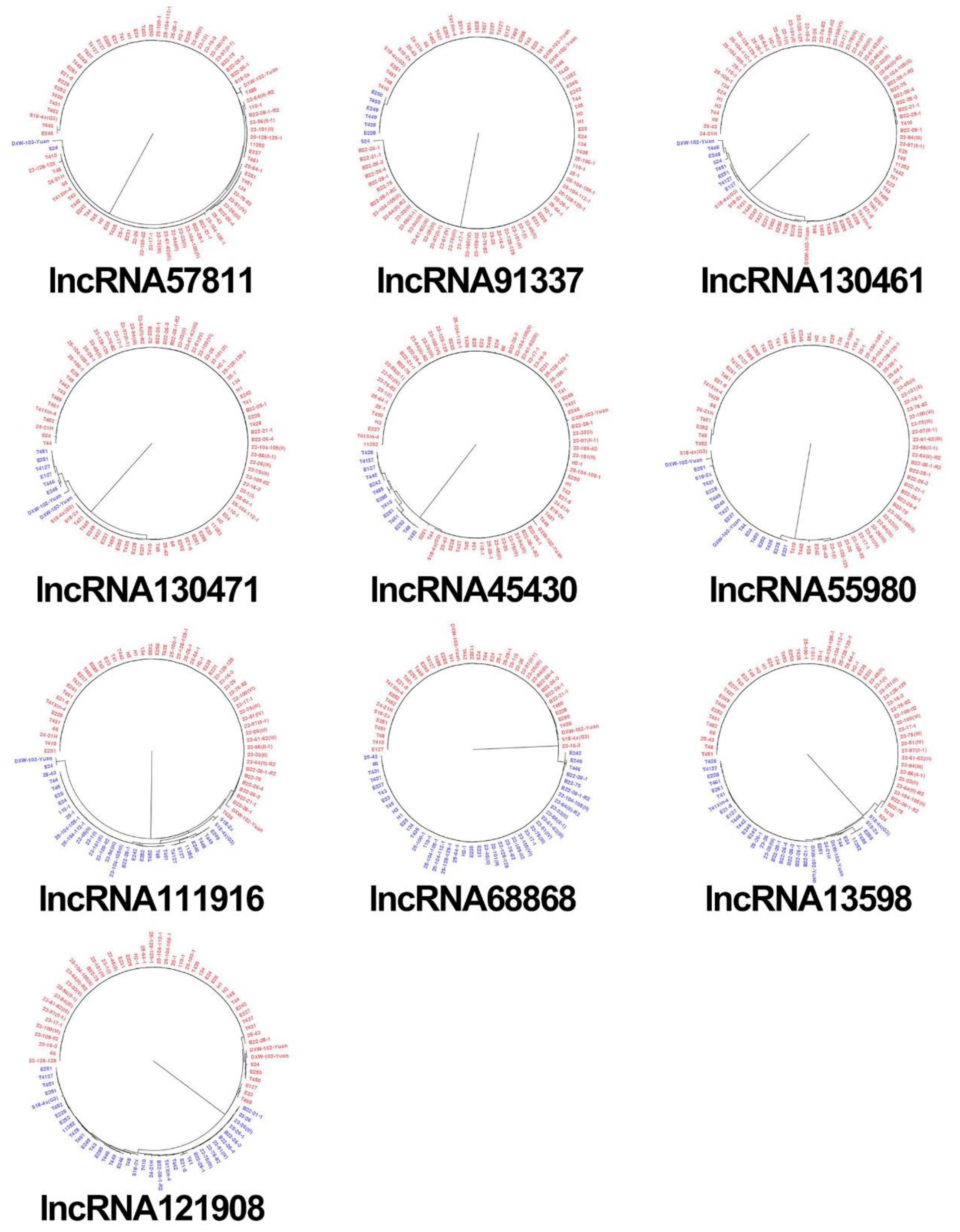

Fig. S4 Phylogenic tree analysis of lncRNAs based on the rice dataset. More than 100 re-sequenced rice lines were included in the rice dataset, including neo-tetraploid, autotetraploid, typical *japonica*/*indica* and wild rice lines. Red and blue font represent the cluster 1 and cluster 2, respectively.

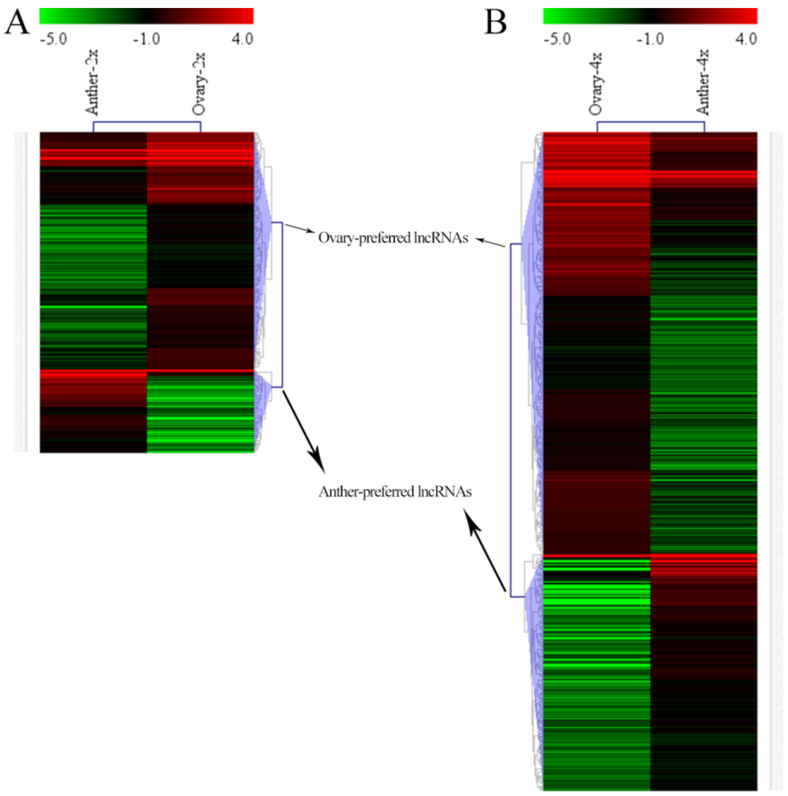

Fig. S5 Anther and ovary-preferred lncRNAs in diploid (A) and autotetraploid rice (B). The hierarchical clustering tree was constructed by MultiExperiment View (version 4.9). The scale bar indicates the relative expression levels of lncRNAs (log2).

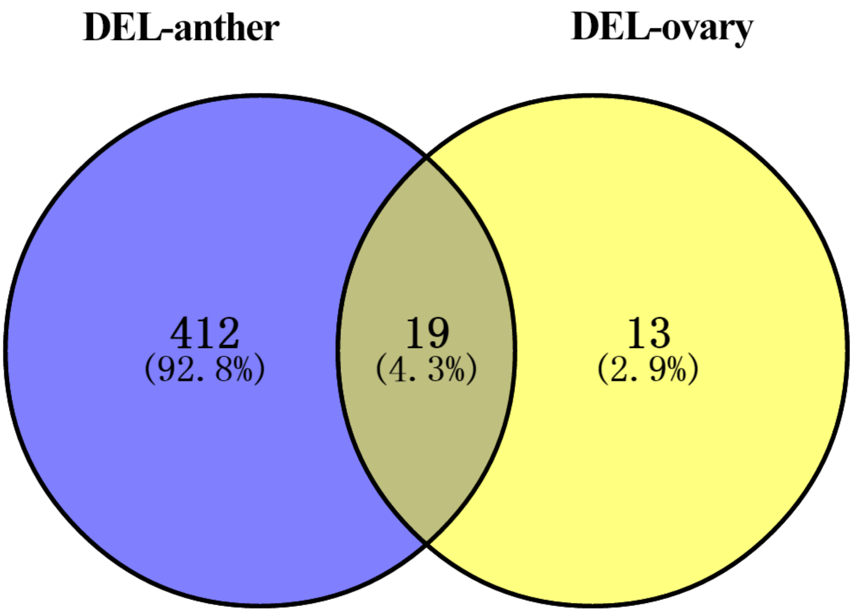

Fig. S6 Venn analysis of DEL-anther and DEL-ovary in autotetraploid rice. DEL-anther/DEL-ovary: differentially expressed lncRNAs in anther/ovary of autotetraploid rice compared to diploid rice.

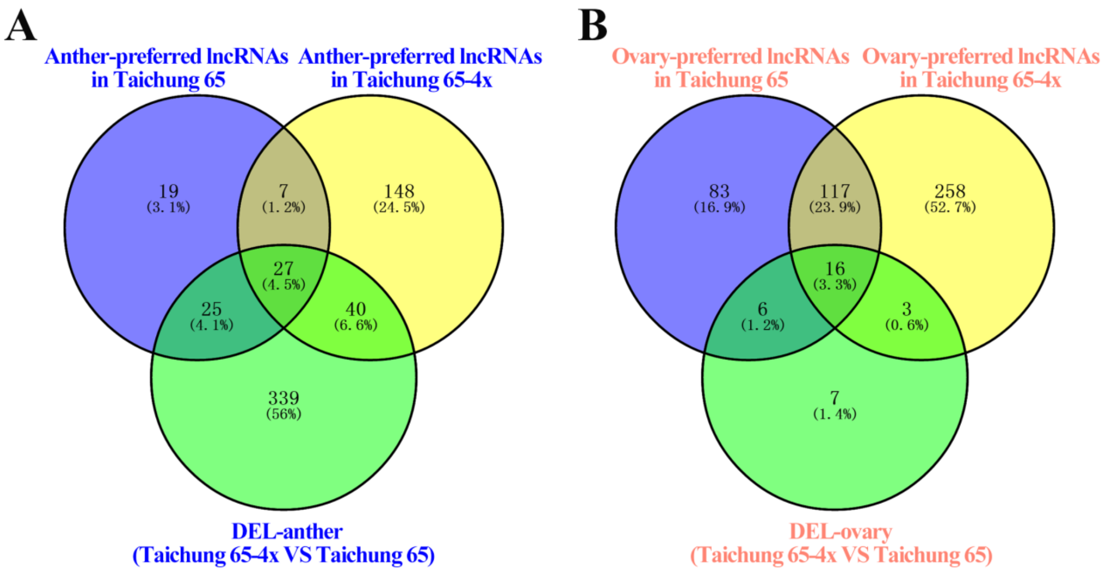

Fig. S7 Classification of differentially expressed lncRNAs in anther (A) and ovary (B) of autotetraploid rice. DEL-anther/ovary represents the differentially expressed lncRNAs in anther/ovary between autotetraploid rice and diploid rice. Type 1 (27 and 16): The DEL overlapped with the co-anther-preferred/co-ovary-preferred lncRNAs of Taichung 65-4x and Taichung 65. Type 2 (25 and 6): The DEL overlapped with anther-preferred/ovary-preferred lncRNAs of Taichung 65. Type 3 (40 and 3): The DEL overlapped with anther-preferred/ovary-preferred lncRNAs of Taichung 65-4x Type 4 (339 and 7): The rest of the DEL in anther/ovary.

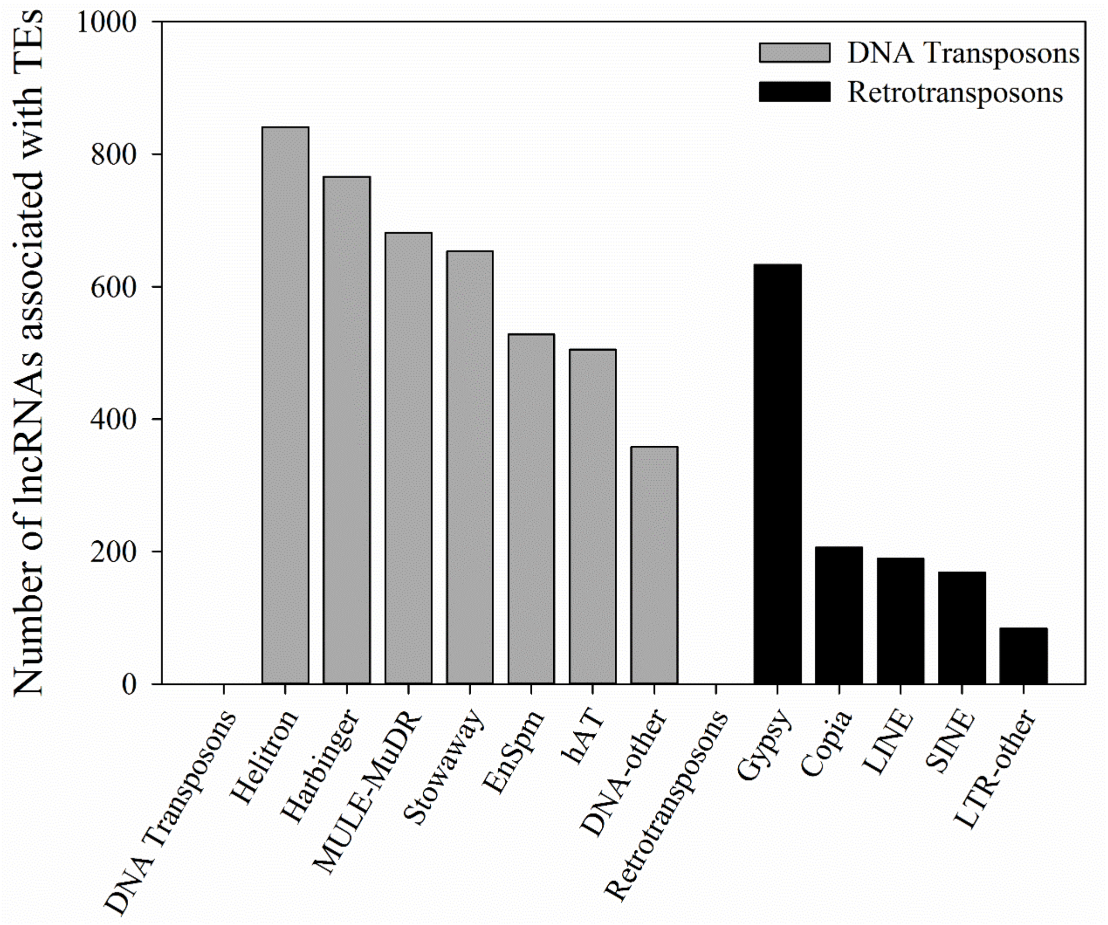

Fig. S8 Transposable elements (TEs) associated with lncRNAs in autotetraploid rice.

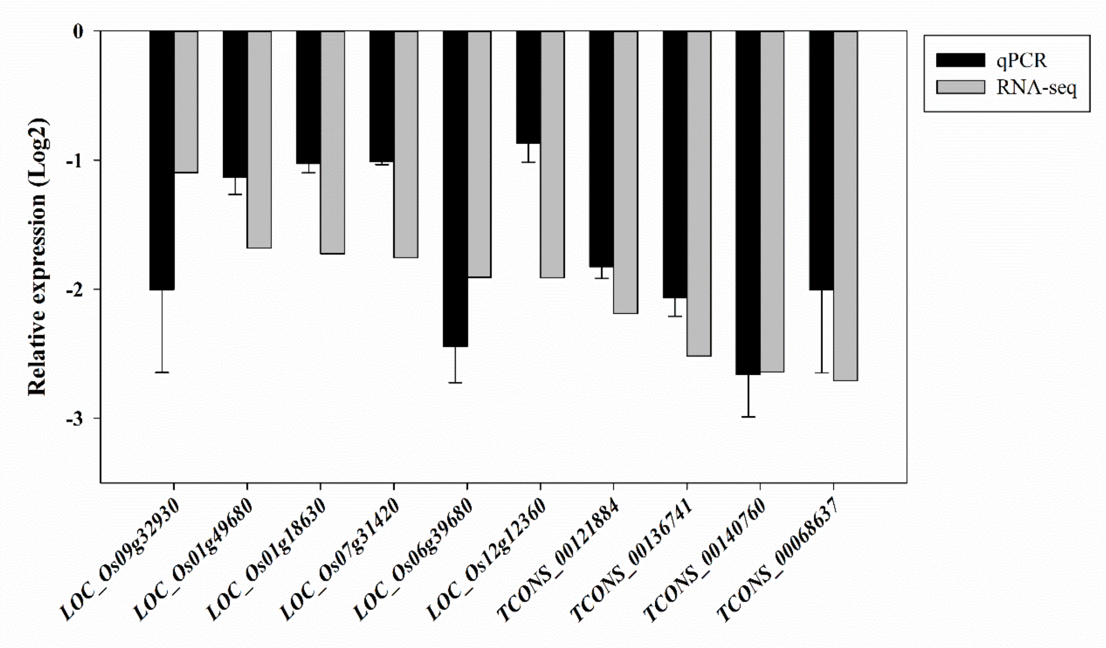

Fig. S9 Validation of the transposable elements (TEs) associated genes and TEs-lncRNAs in autotetraploid compared to diploid rice. The x- and y-axis represent the TEs/TEs-lncRNAs and relative expression levels, respectively. Error bars represent the standard deviation (SD) of three biological replicates.

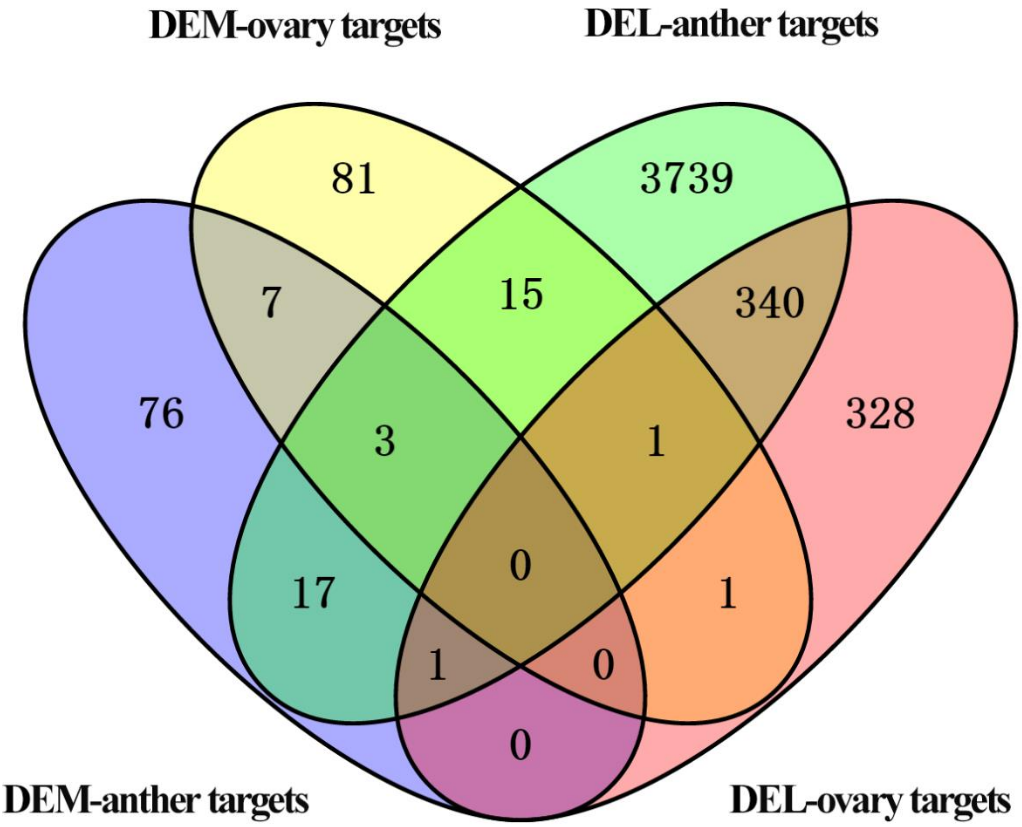

Fig. S10 Venn analysis of the predicted targets of differentially expressed lncRNAs (DEL) and differentially expressed miRNAs (DEM). DEM-anther: DEM in autotetraploid rice anther compared to diploid rice. DEM-ovary: DEM in autotetraploid rice ovary compared to diploid rice. DEL-anther: DEL in autotetraploid rice anther compared to diploid rice. DEL-ovary: DEL in autotetraploid rice ovary compared to diploid rice.

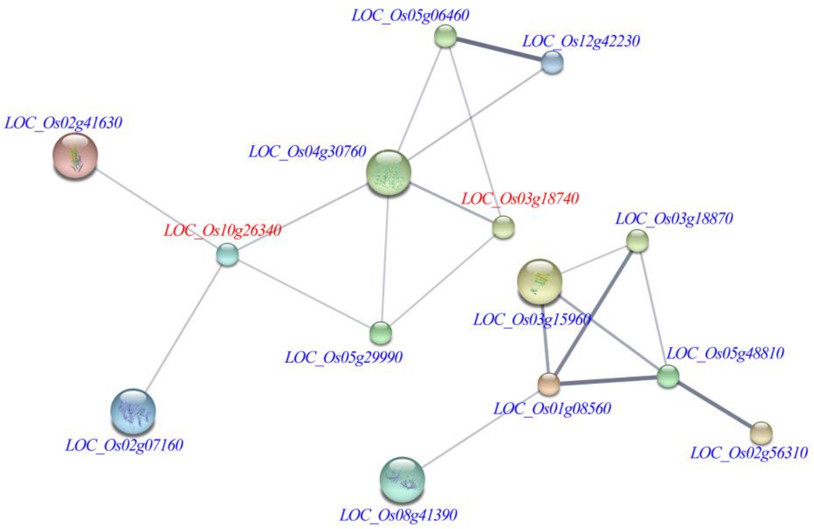

Fig. S11 Protein-protein interactions between the EMC meiosis-related targets of differentially expressed lncRNAs. Red and blue font represent the up- and down-regulated differentially expressed targets in autotetraploid rice, respectively. The DEL regulators of targets are shown in Additional file 4:Table S18.

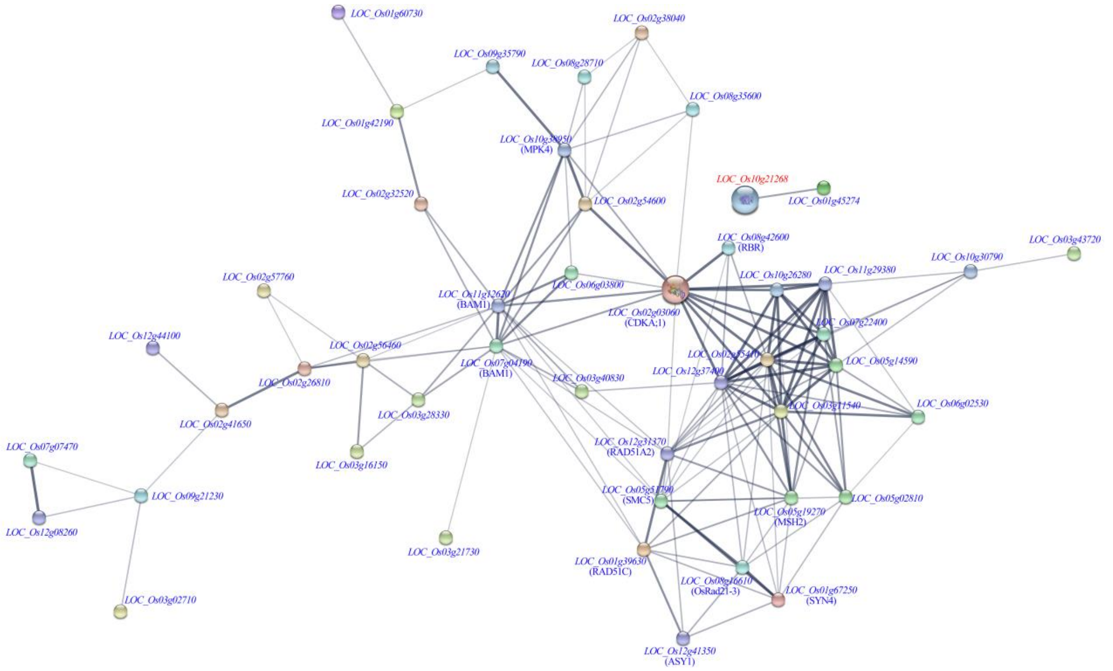

Fig. S12 Protein-protein interactions between the PMC meiosis-related targets of differentially expressed lncRNAs. Red and blue font represent the up- and down-regulated differentially expressed targets in autotetraploid rice. The DEL regulators of targets are presented in Additional file 4: Table S17.

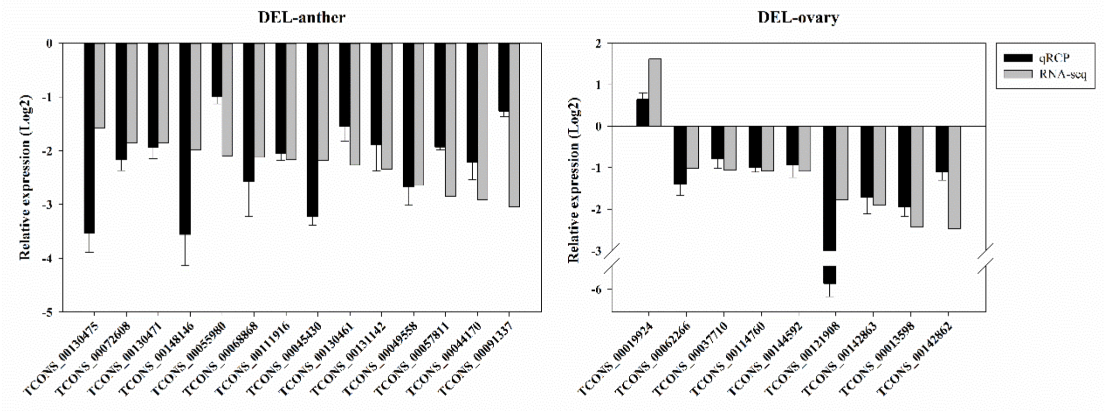

Fig. S13 Validation of the differentially expressed lncRNAs (DEL) in autotetraploid and diploid rice. The x- and y-axis represent the lncRNAs and relative expression levels, respectively. Error bars represent the standard deviation (SD) of three biological replicates. DEL-anther/ovary represents the differentially expressed lncRNAs in anther/ovary between autotetraploid rice and diploid rice.

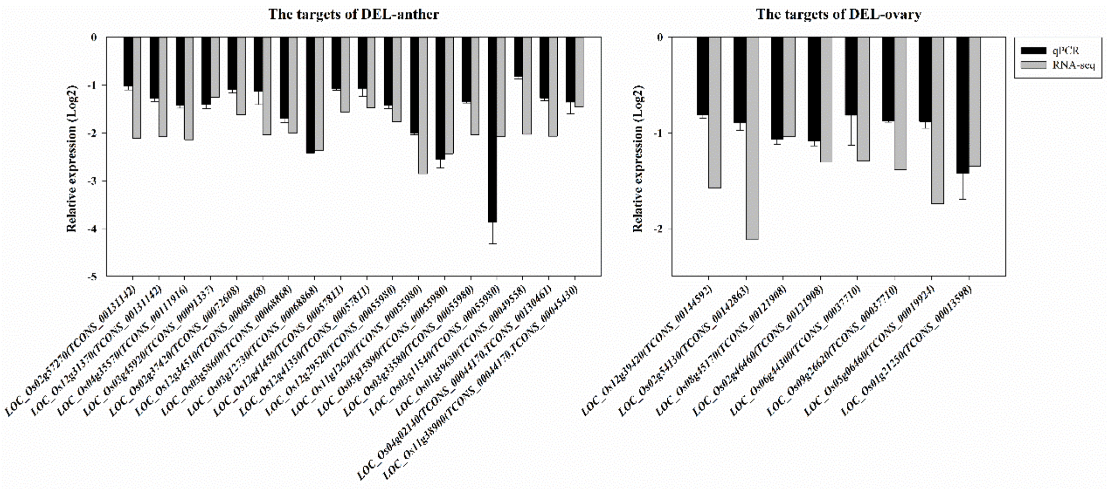

Fig. S14 Validation of the targets of differentially expressed lncRNAs in autotetraploid and diploid rice. The x- and y-axis represent the targets of DEL and relative expression levels, respectively. Error bars represent the standard deviation (SD) of three biological replicates.

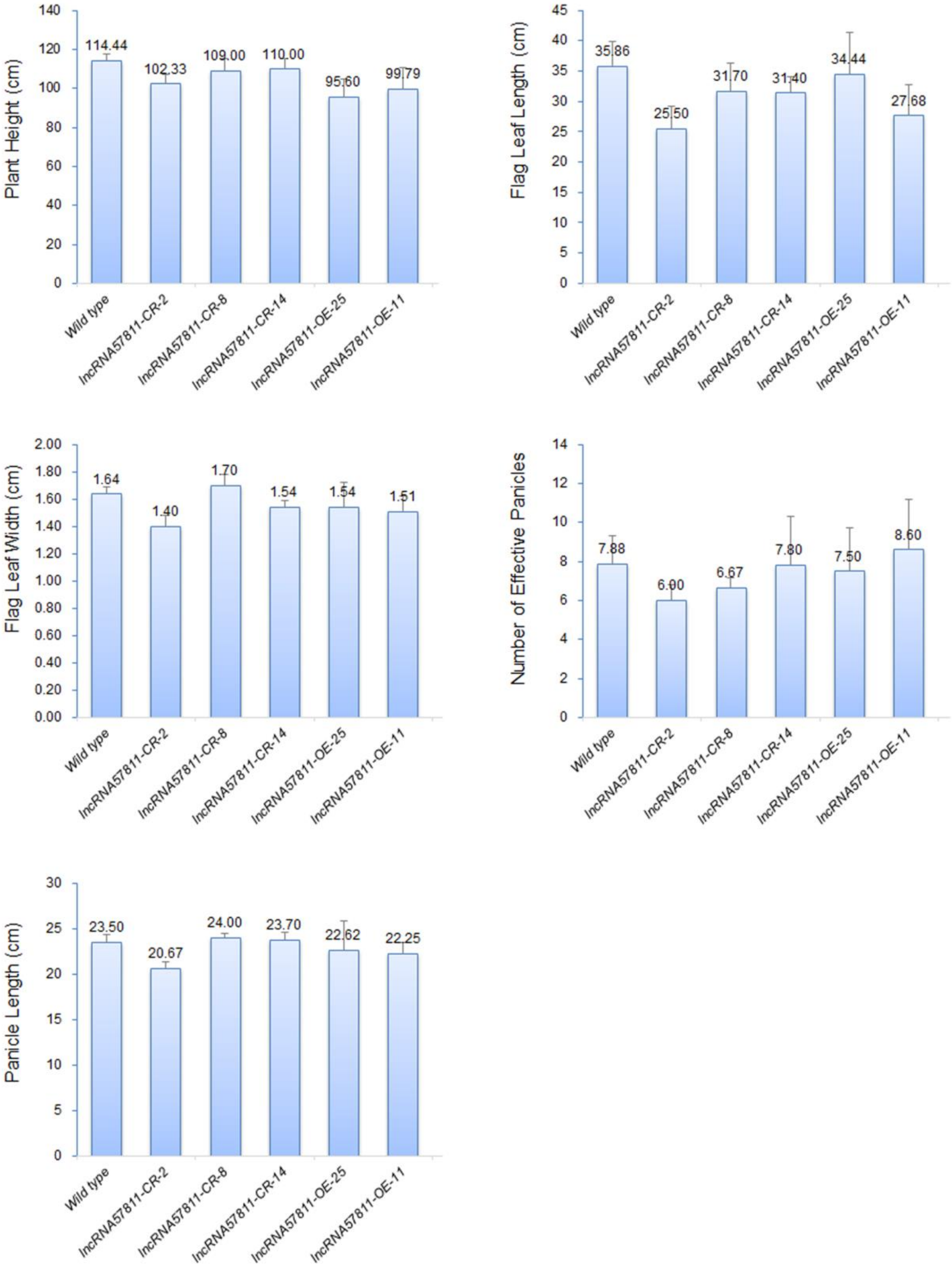

Fig. S15 Agronomic traits of wild type and mutants in T1 generation.

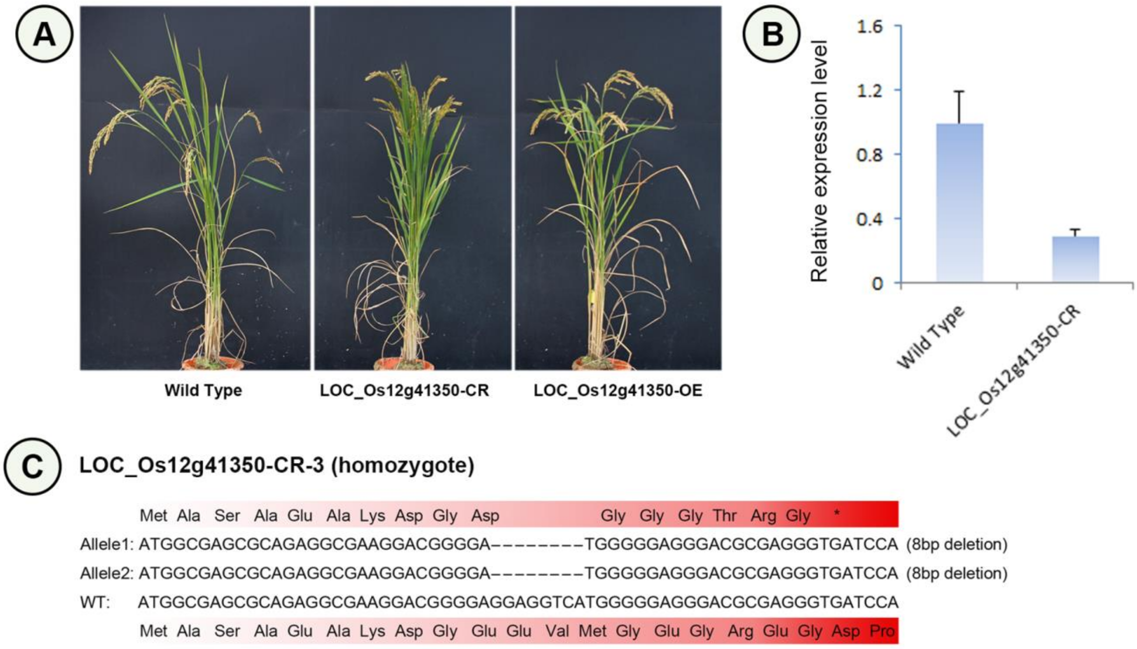

Fig. S16 Phenotypes of *LOC_Os12g41350*-CR and *LOC_Os12g41350*-OE in T1 generation. (A) Comparison between the wild-type plant and *LOC_Os12g41350* mutant plant after heading. (B) *LOC_Os12g41350*-CR mutants have low expression of *LOC_Os12g41350* compared to wild type. (C) Genotype of *LOC_Os12g41350*-CR lines.
